# Supplementary material for: GrandQC: A comprehensive solution to quality control problem in digital pathology
Source: Nat Commun. 2024 Dec 16;15:10685. doi: 10.1038/s41467-024-54769-y (PMC11649692; doi:10.1038/s41467-024-54769-y)
Supplement: Supplementary file 1 — Supplementary Information [file 41467_2024_54769_MOESM1_ESM.pdf]

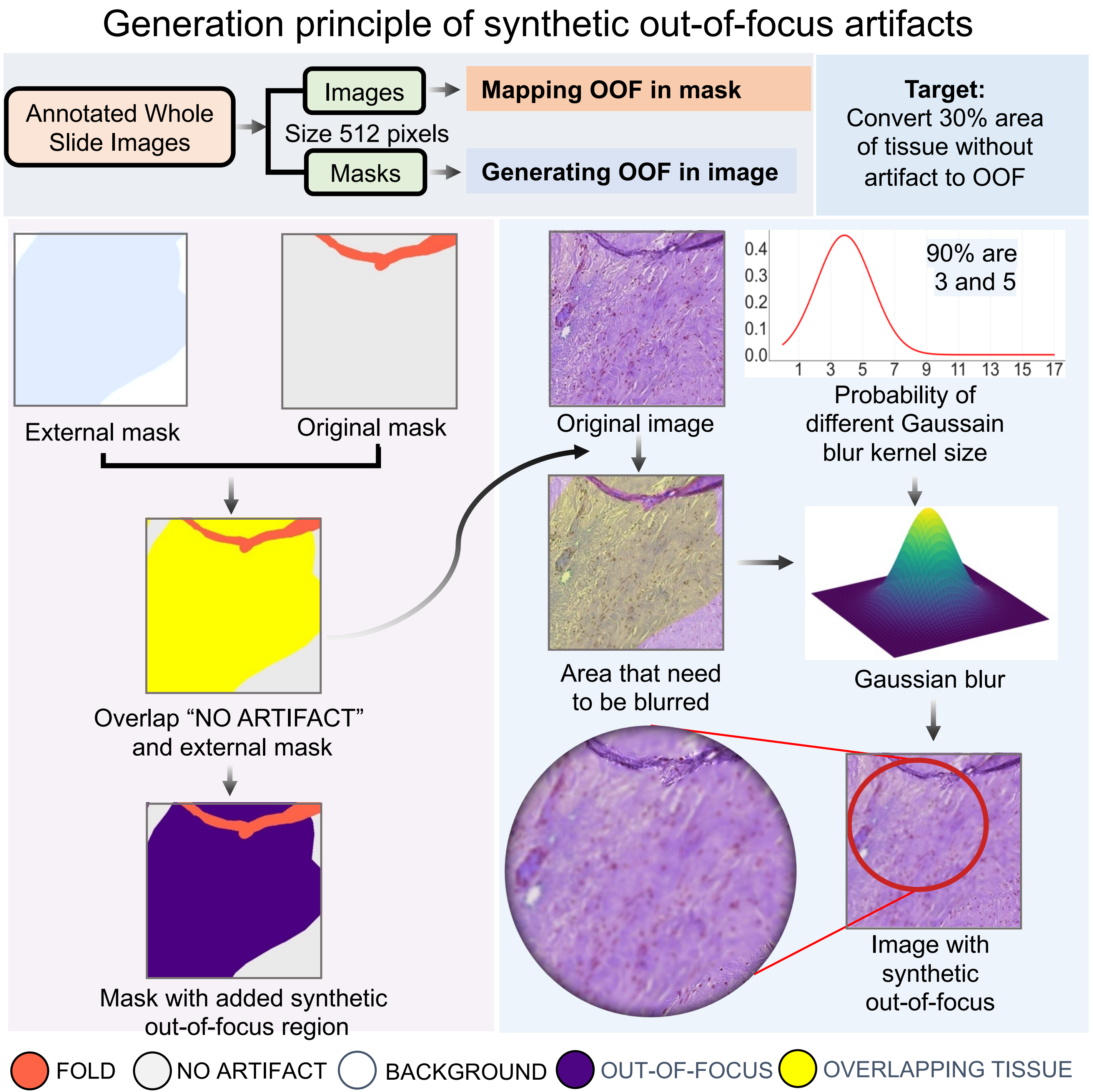

Supplementary Fig. 1 Principle of generation of synthetic out-of-focus (OOF) artifacts used for training dataset.

Examples of whole-slide images used for GrandQC performance test

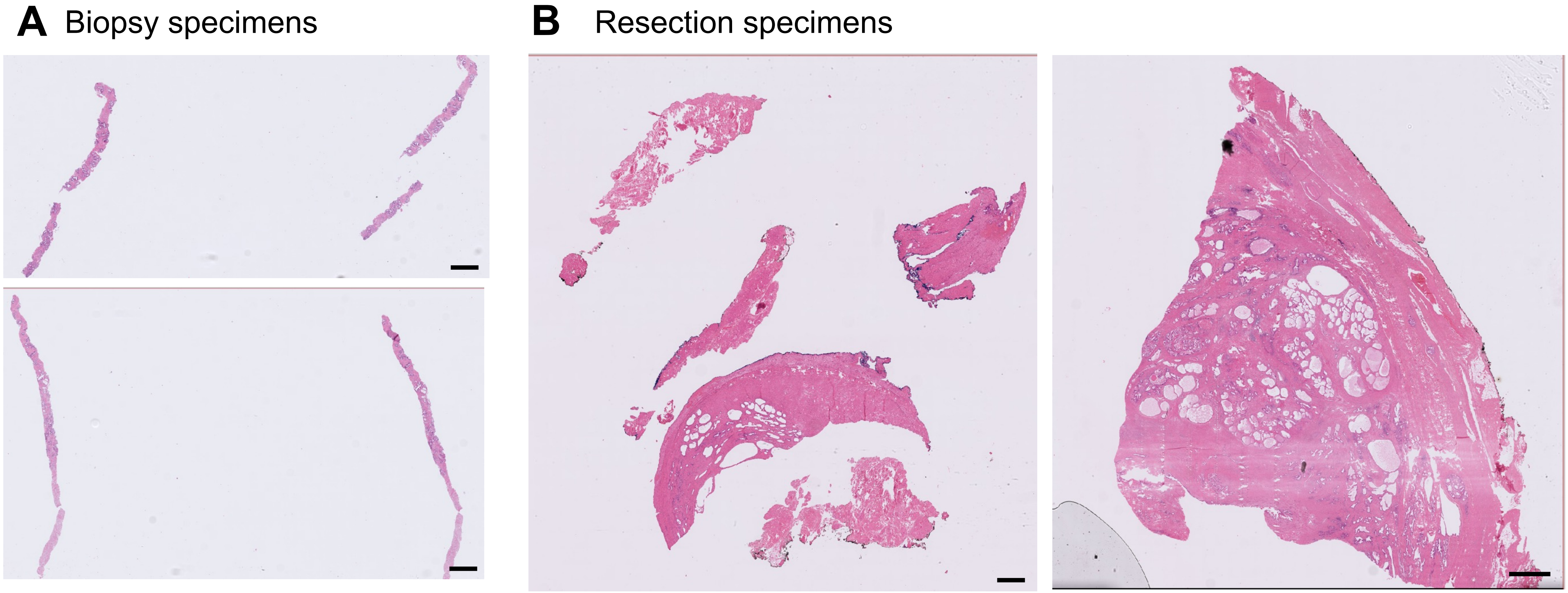

Supplementary Fig. 2 Examples of whole-slide images used for GrandQC performance test. All scale bars are 2 mm.

Performance of the 5x, 7x, 10x version of GrandQC artifact detection module

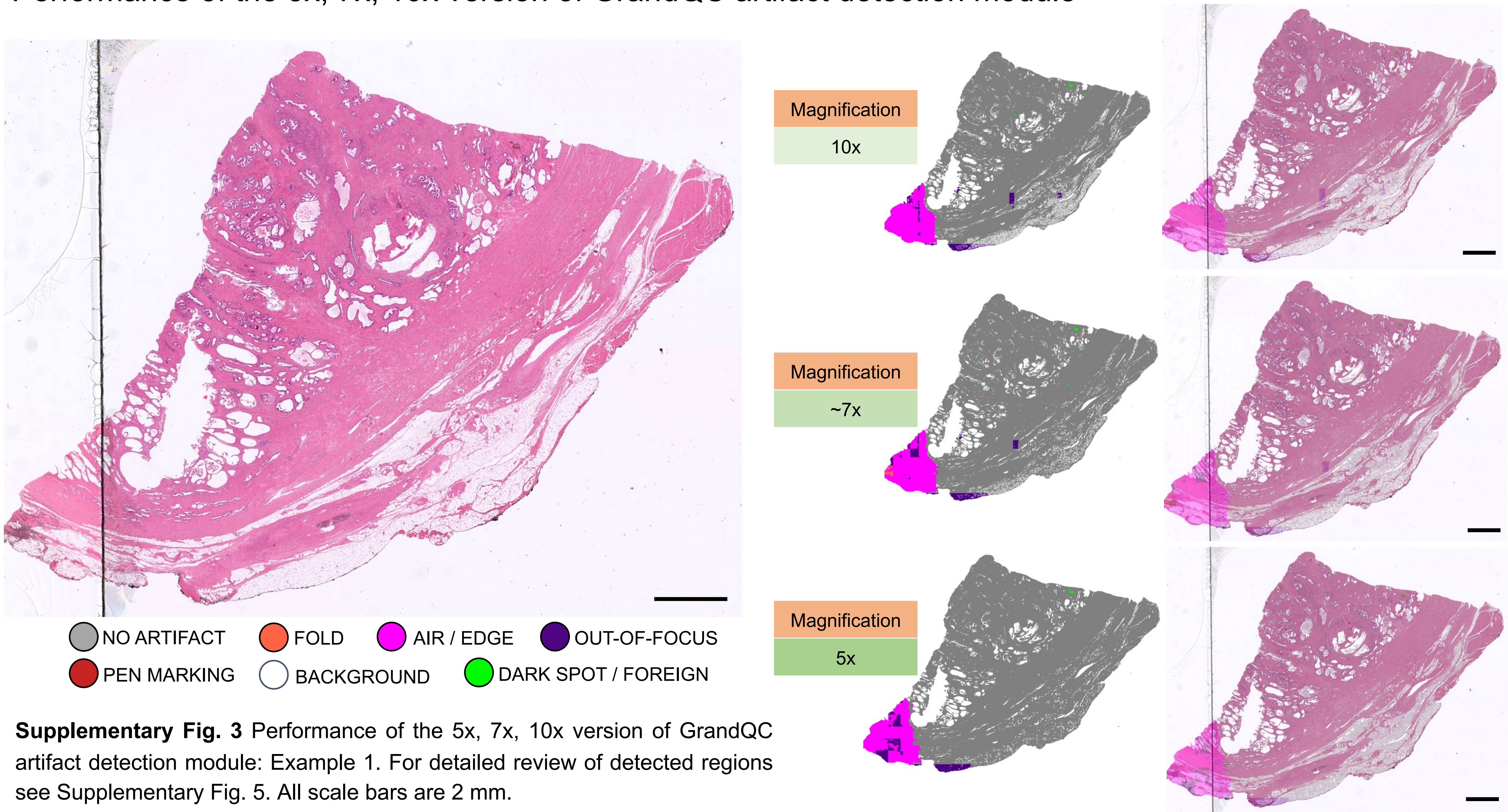

Supplementary Fig. 3 Performance of the 5x, 7x, 10x version of GrandQC artifact detection module: Example 1. For detailed review of detected regions see Supplementary Fig. 5. All scale bars are 2 mm.

Performance of the 5x, 7x, 10x version of GrandQC artifact detection module

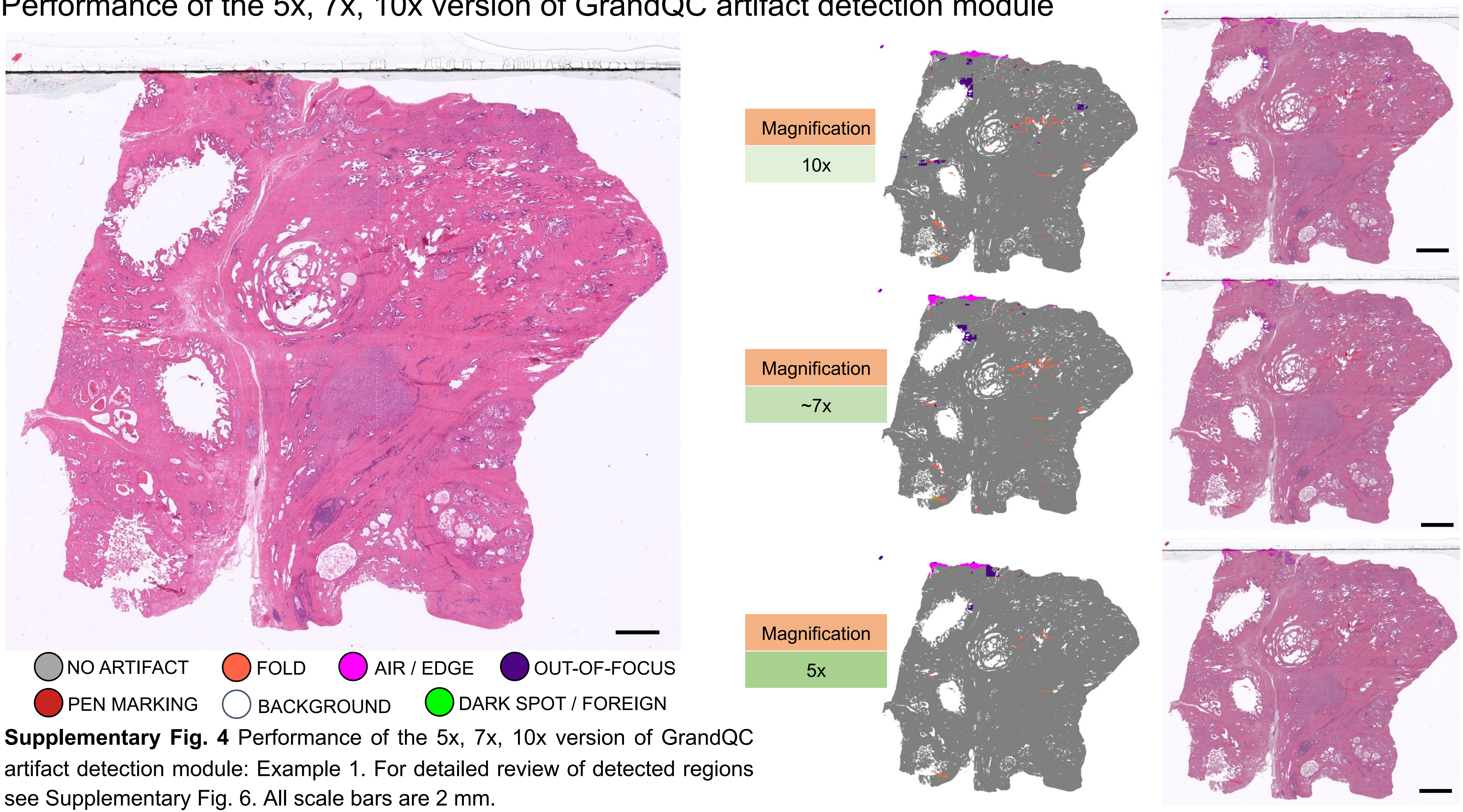

Supplementary Fig. 4 Performance of the 5x, 7x, 10x version of GrandQC artifact detection module: Example 1. For detailed review of detected regions see Supplementary Fig. 6. All scale bars are 2 mm.

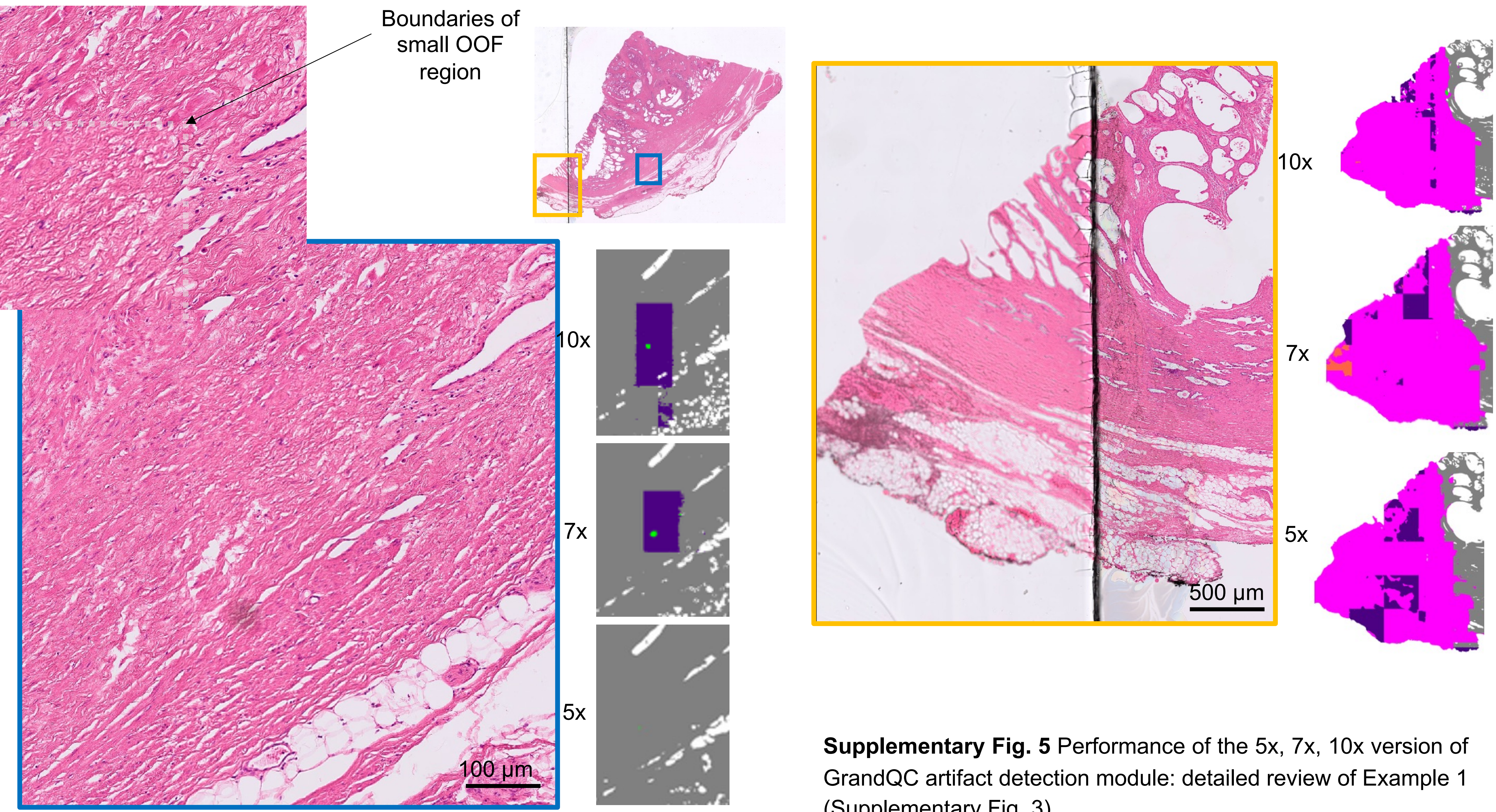

Supplementary Fig. 5 Performance of the 5x, 7x, 10x version of GrandQC artifact detection module: detailed review of Example 1 (Supplementary Fig. 3).

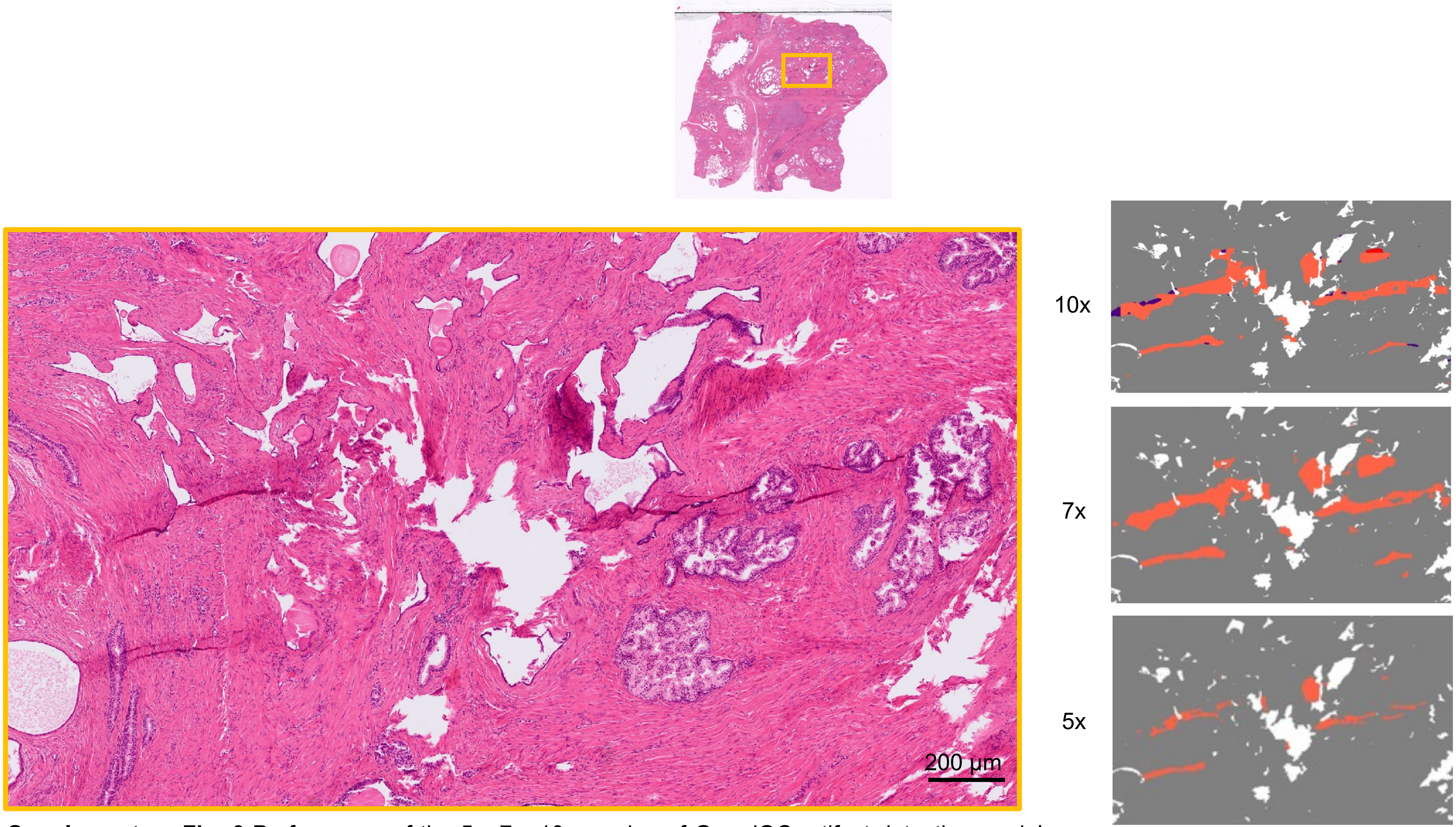

Supplementary Fig. 6 Performance of the 5x, 7x, 10x version of GrandQC artifact detection module: detailed review of Example 2 (Supplementary Fig. 4).

Focal misclassification of highly pigmented regions in malignant melanoma cases as air bubble/edge artifact

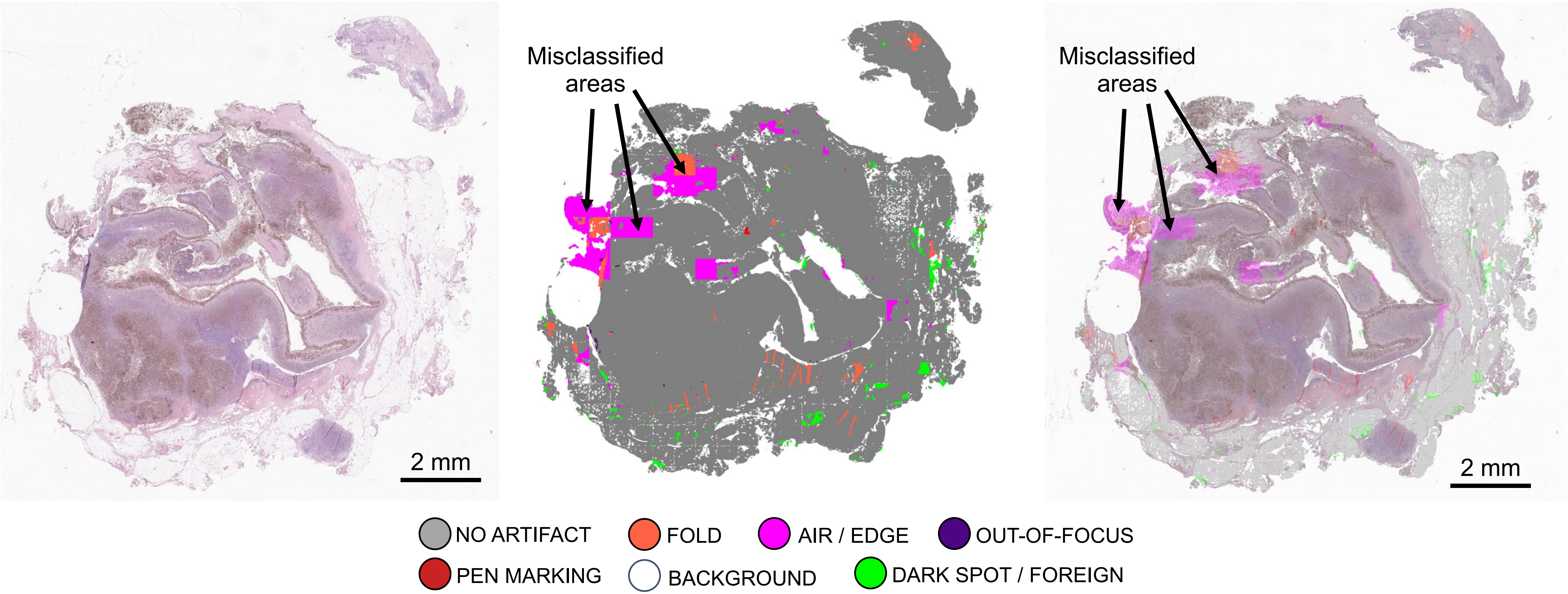

Supplementary Fig. 7 Examples of pigmented areas misclassified as air bubble artifact in whole slide images of highly pigmented malignant melanoma.

A GrandQC: downstream performance, use case 1

Different artifact types as a reason for false-positive tumor detection

ROI, n	Fold	Dark Spot & Foreign object	Penmarking	Glass edge & Air	Out-of-focus
**Lung (n=105)**	14	3	2	-	-
**Colon (n=33)**	4	2	-	-	1

B GrandQC: downstream performance, use case 1

Preventing false positive tumor misclassifications in regions with benign tissue

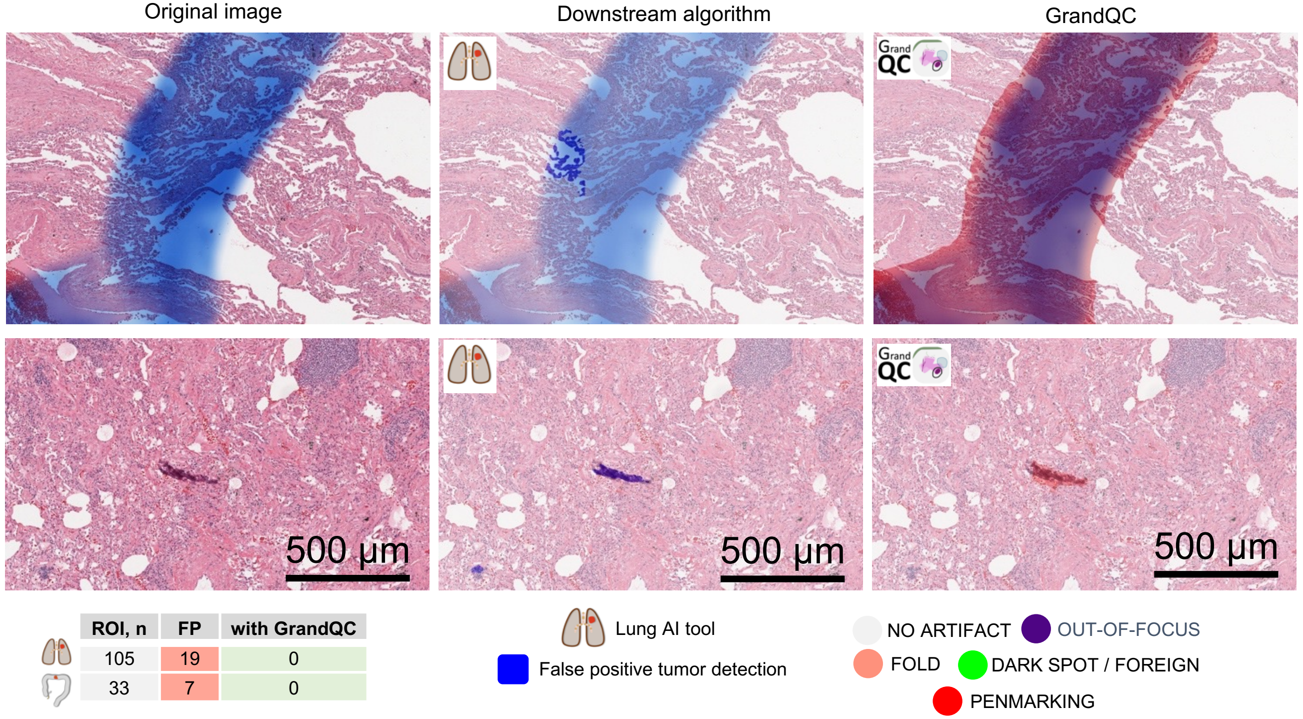

Supplementary Fig. 8 GrandQC enhances the performance of downstream diagnostic multi-class tissue segmentation algorithms: preventing false positive tumor classifications in benign tissue regions. A. Structure of regions-of-interest is presented as well as which artifact type was responsible for misclassification. B. Representative examples demonstrate how the detection and masking of artifacts help prevent false positive misclassifications in regions of interests containing benign tissue. Comment: These regions were analyzed using previously developed multi-class tissue segmentation algorithms for lung and colorectal cancer, respectively.

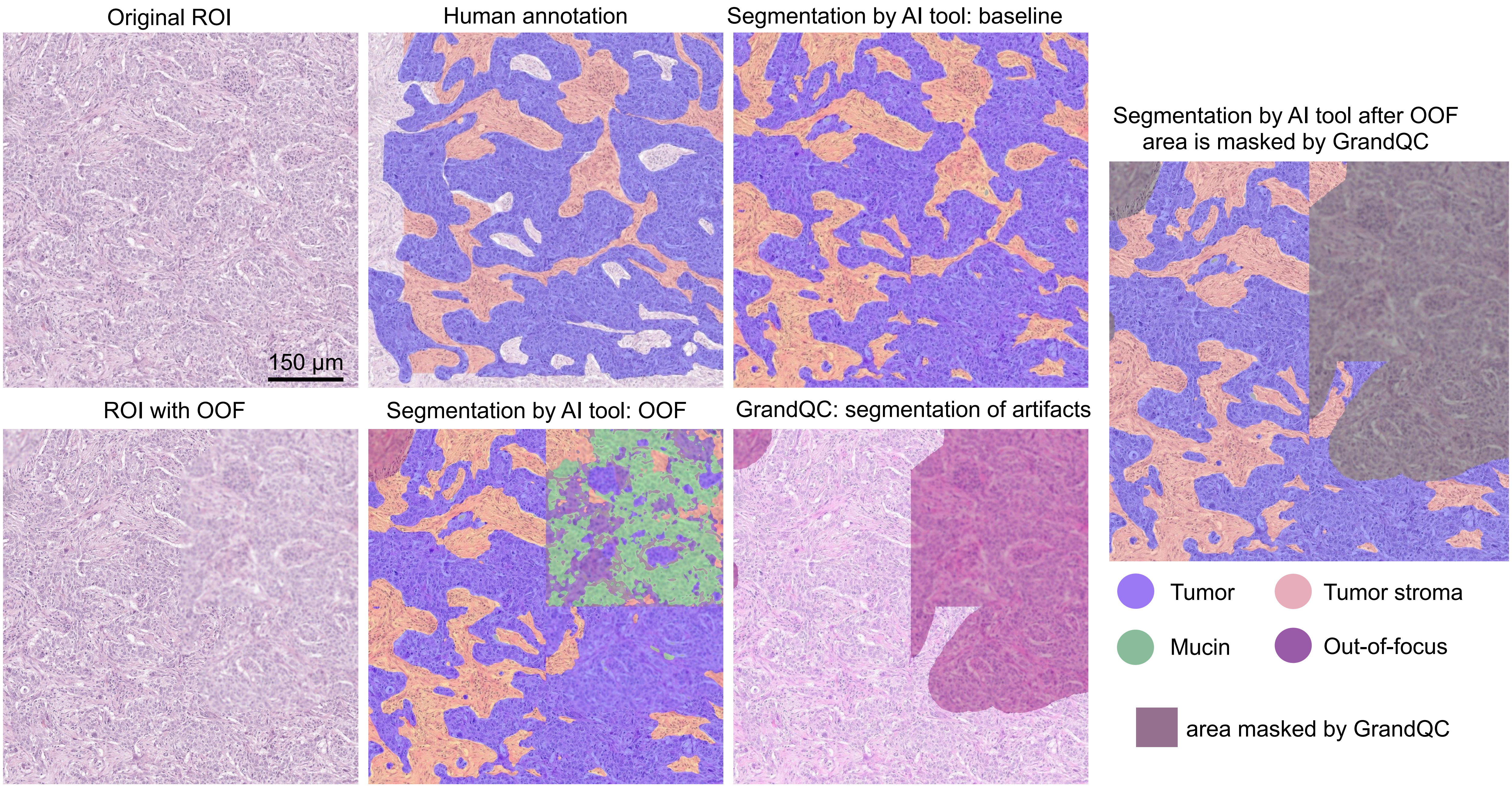

Supplementary Fig. 9 Impact of out-of-focus artifact on downstream tasks – Lung AI tool (Kludt et al. Cell Rep Medicine 2024)

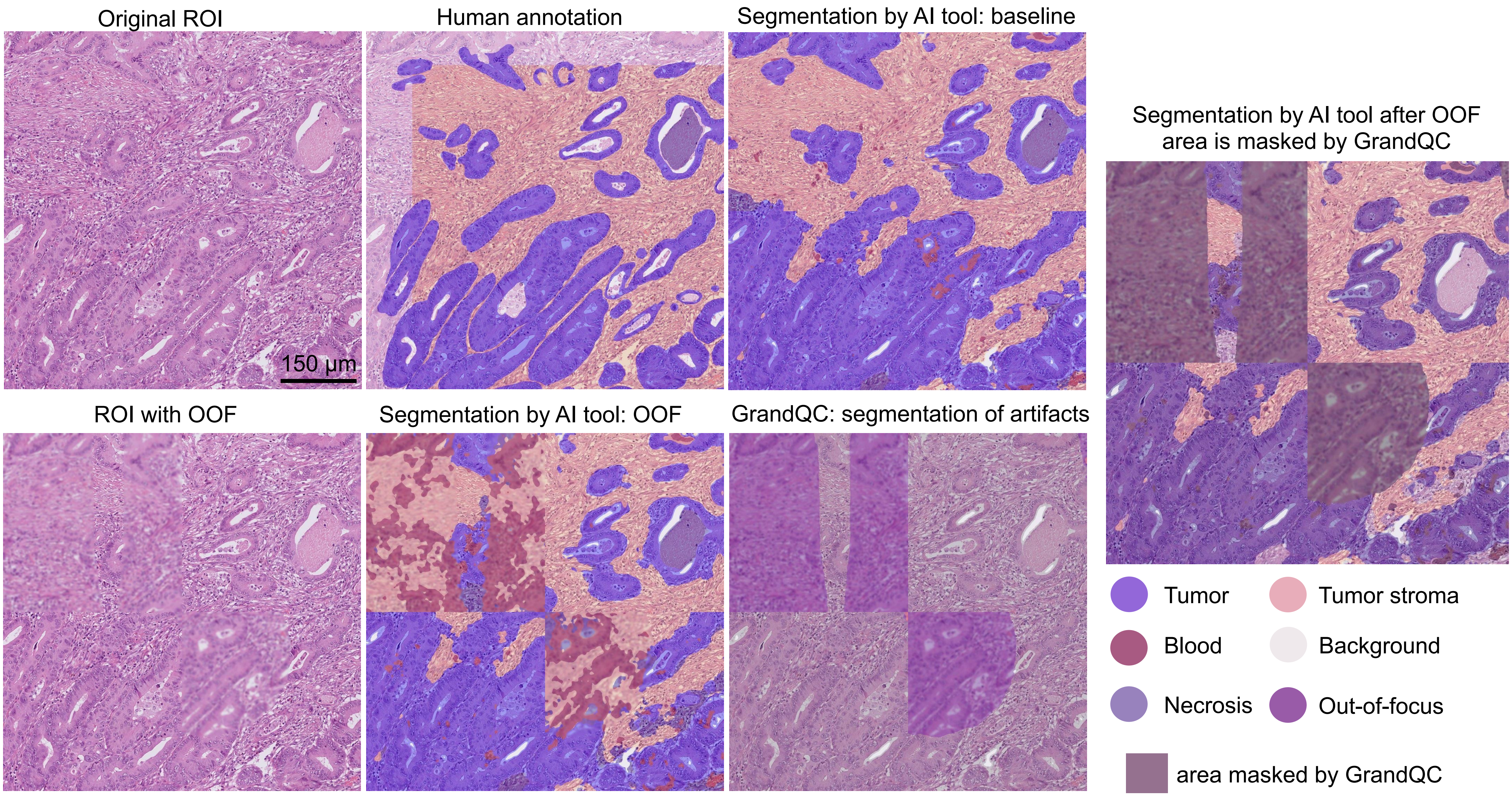

Supplementary Fig. 10 Impact of out-of-focus artifact on downstream tasks – Colorectal AI tool (Griem et al. Mod Path 2023)

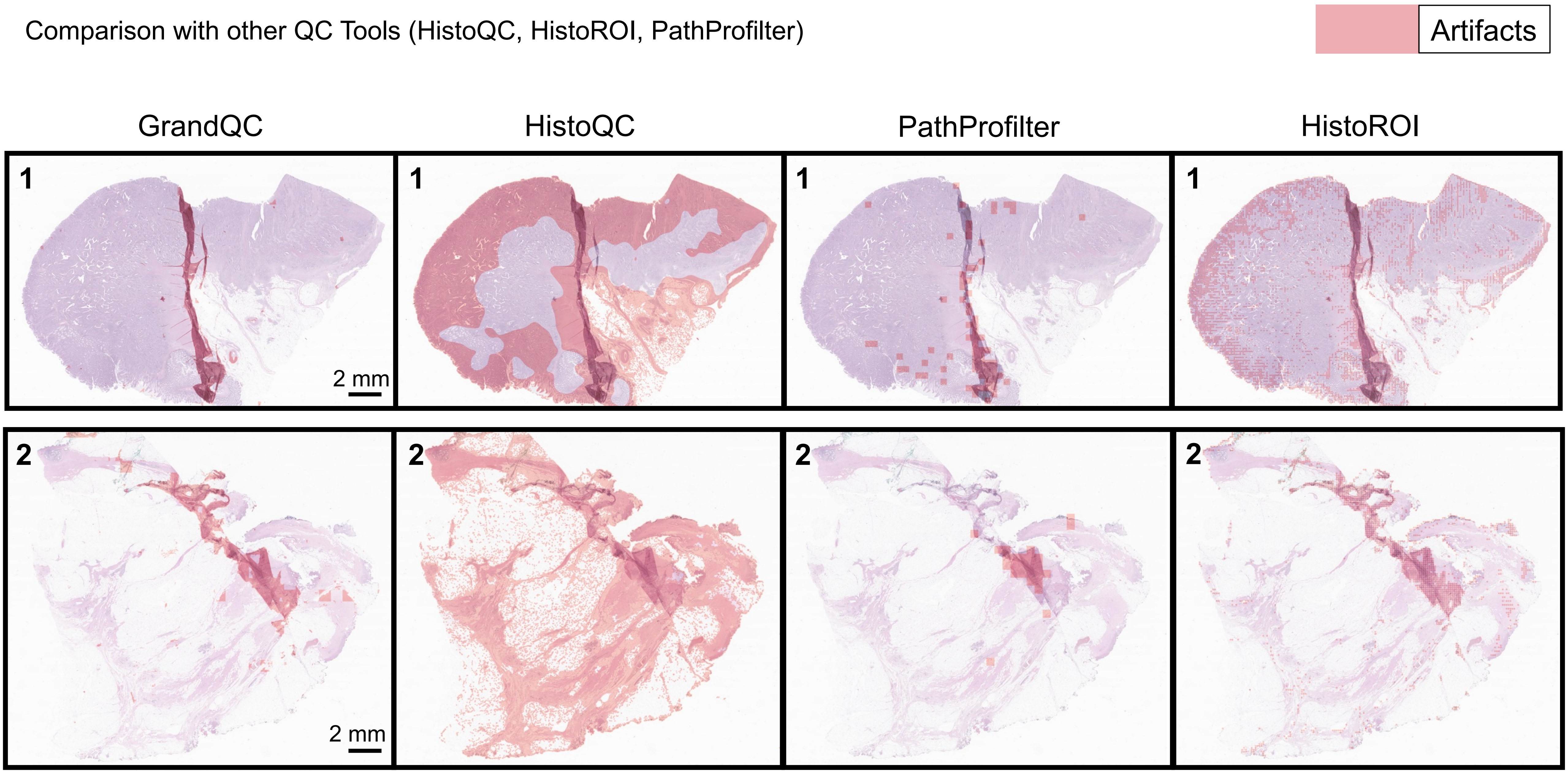

Supplementary Fig. 11 Comparison of GrandQC with three open-source tools for quality control (HistoQC, PathProfiler, HistoROI). Two whole-slide images are presented as examples (for original images in higher resolution see Supplementary Fig. 15-25). Red color corresponds to artificially changed areas detected.

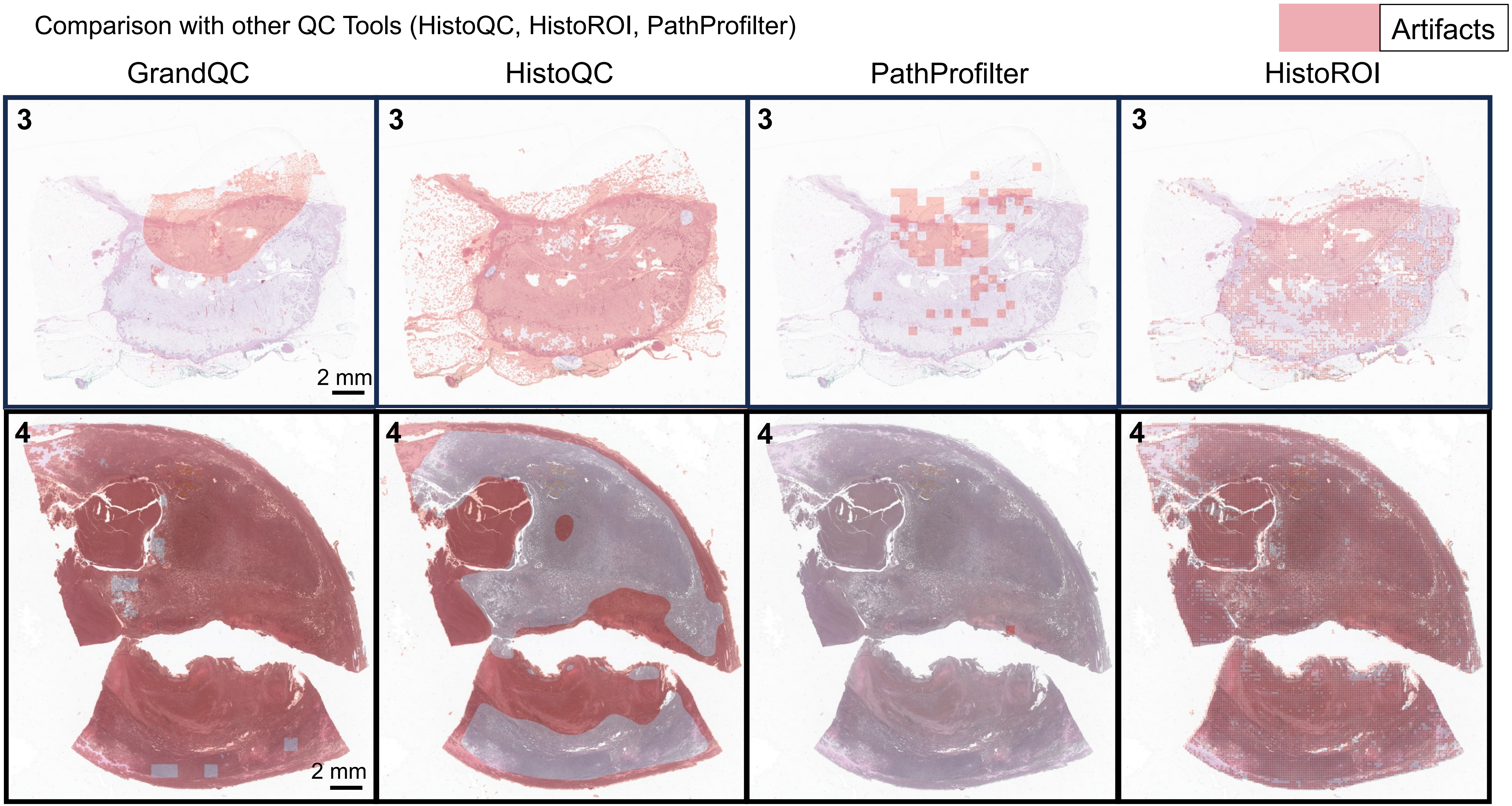

Supplementary Fig. 12 Comparison of GrandQC with three open-source tools for quality control (HistoQC, PathProfiler, HistoROI). Two whole-slide images are presented as examples (for original images in higher resolution see Supplementary Fig. 15-25). Red color = artificially changed areas.

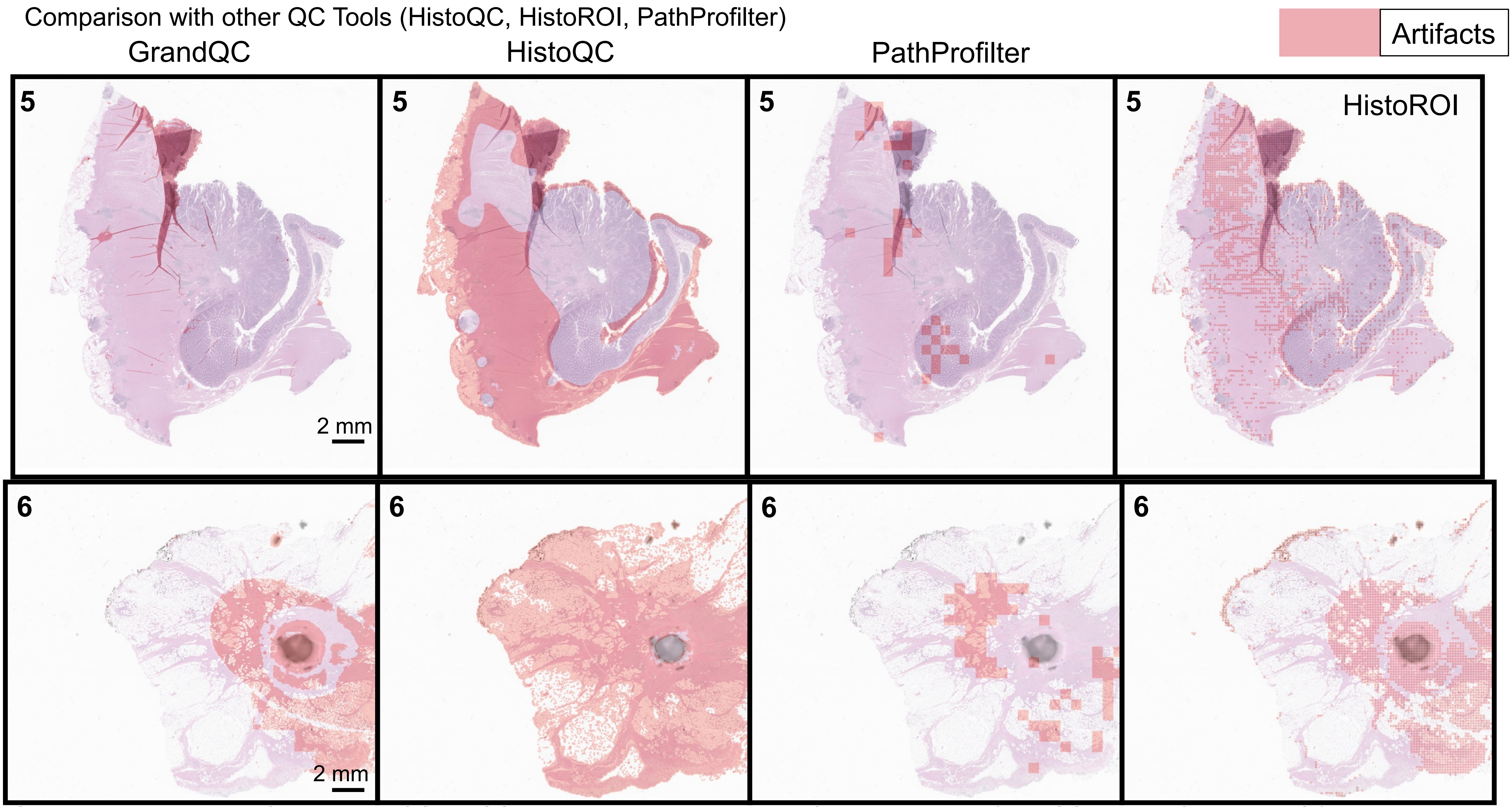

Supplementary Fig. 13 Comparison of GrandQC with three open-source tools for quality control (HistoQC, PathProfiler, HistoROI). Two whole-slide images are presented as examples (for original images in higher resolution see Supplementary Fig. 15-25). Red color = artificially changed areas.

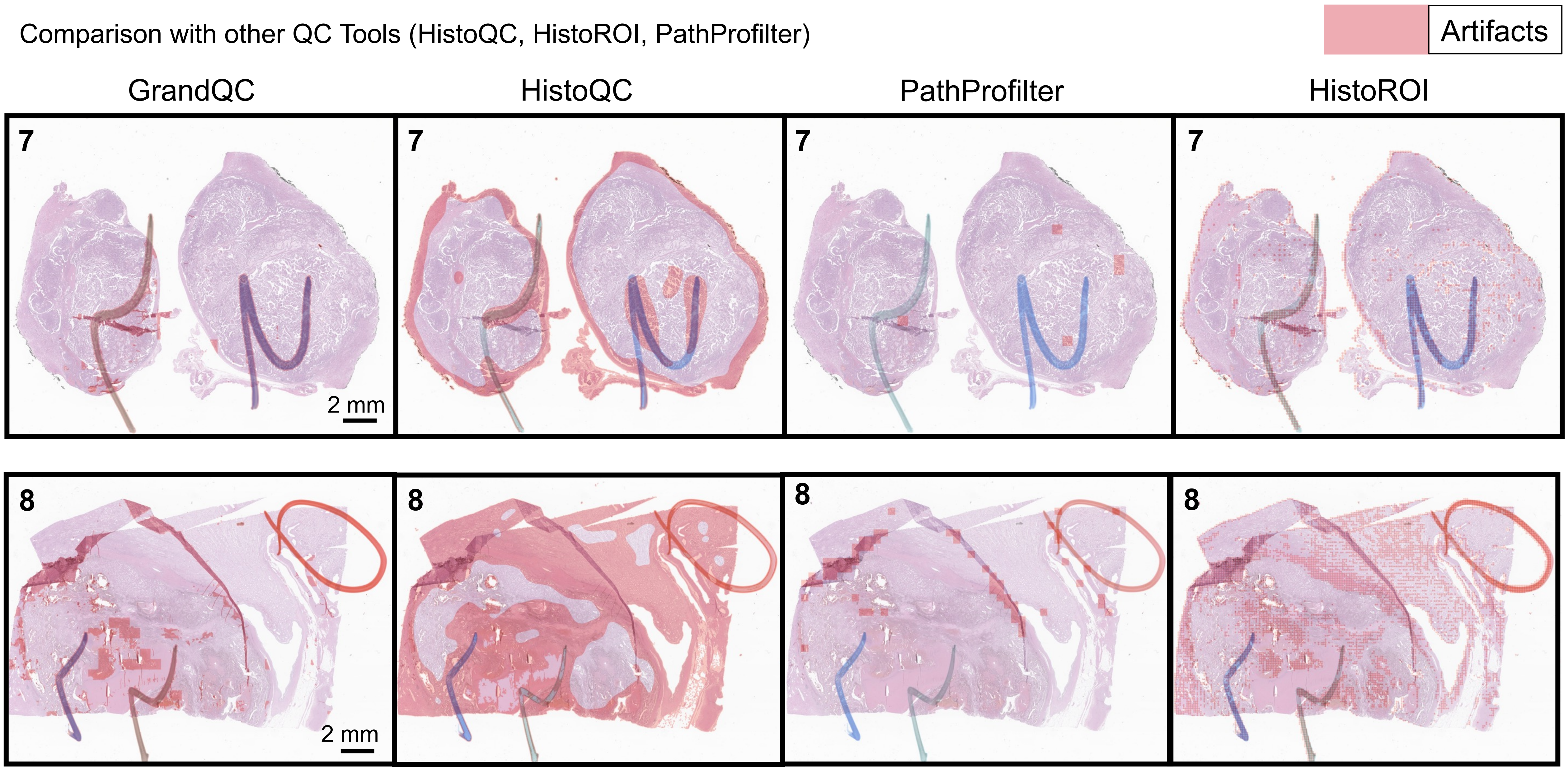

Supplementary Fig. 14 Comparison of GrandQC with three open-source tools for quality control (HistoQC, PathProfiler, HistoROI). Two whole-slide images are presented as examples (for original images in higher resolution see Supplementary Fig. 15-25). Red color = artificially changed areas.

Comparison with other QC Tools (HistoQC, HistoROI, PathProfilter)

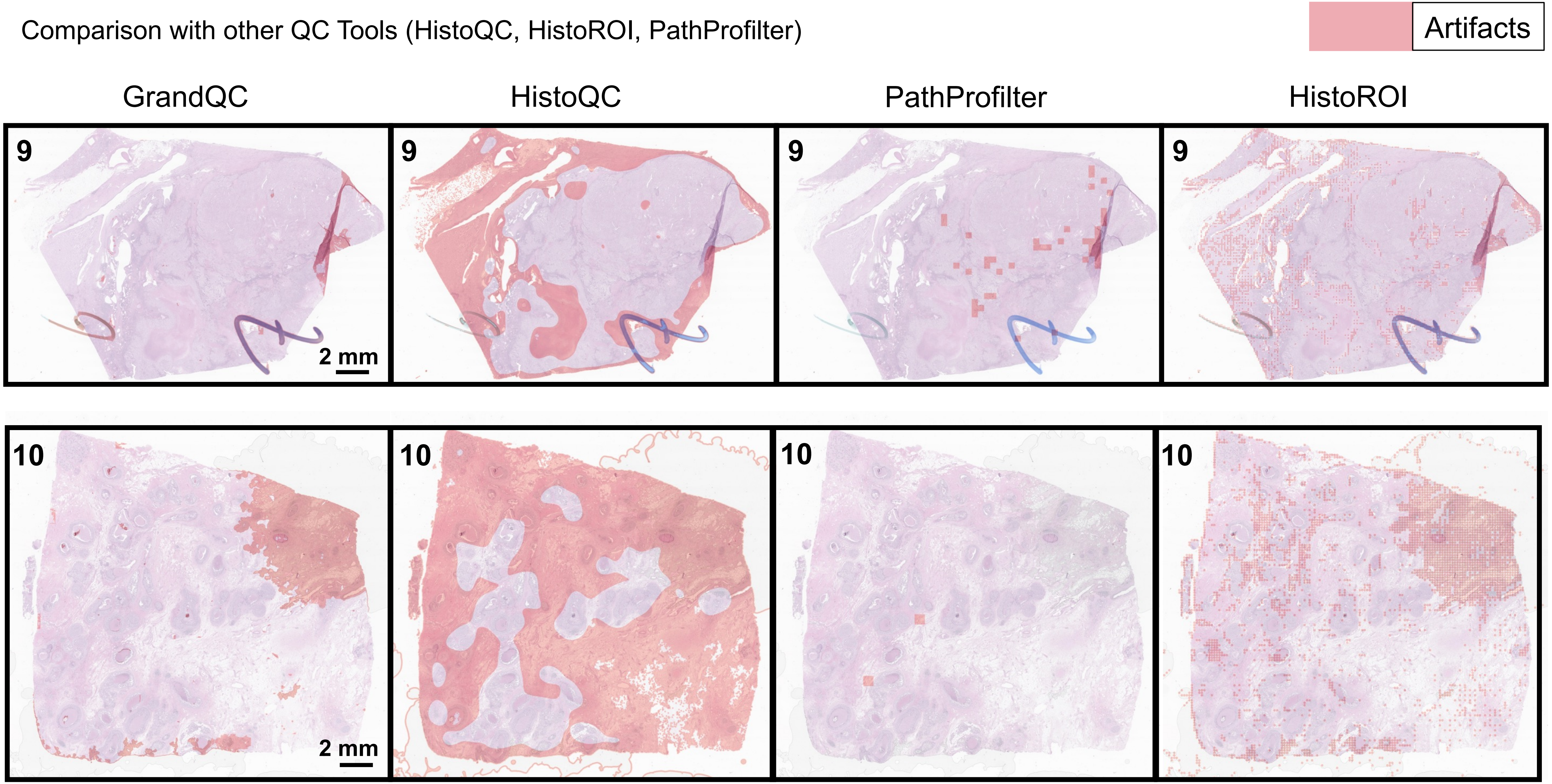

Supplementary Fig. 15 Comparison of GrandQC with three open-source tools for quality control (HistoQC, PathProfiler, HistoROI). Two whole-slide images are presented as examples (for original images in higher resolution see Supplementary Fig. 15-25). Red color = artificially changed areas.

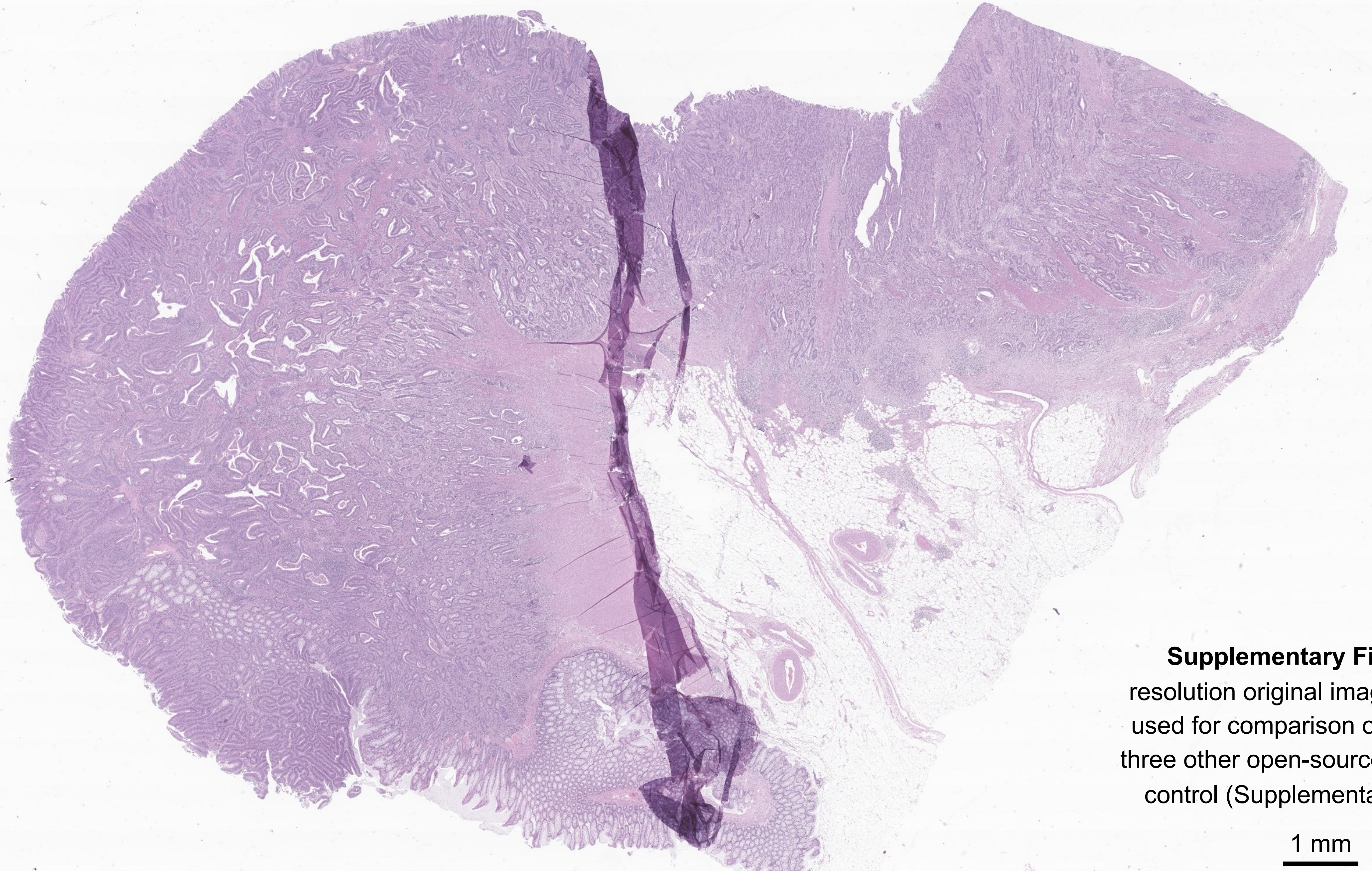

Supplementary Fig. 16 Higher resolution original images of the slides used for comparison of GrandQC with three other open-source tools for quality control (Supplementary Fig. 11-15).

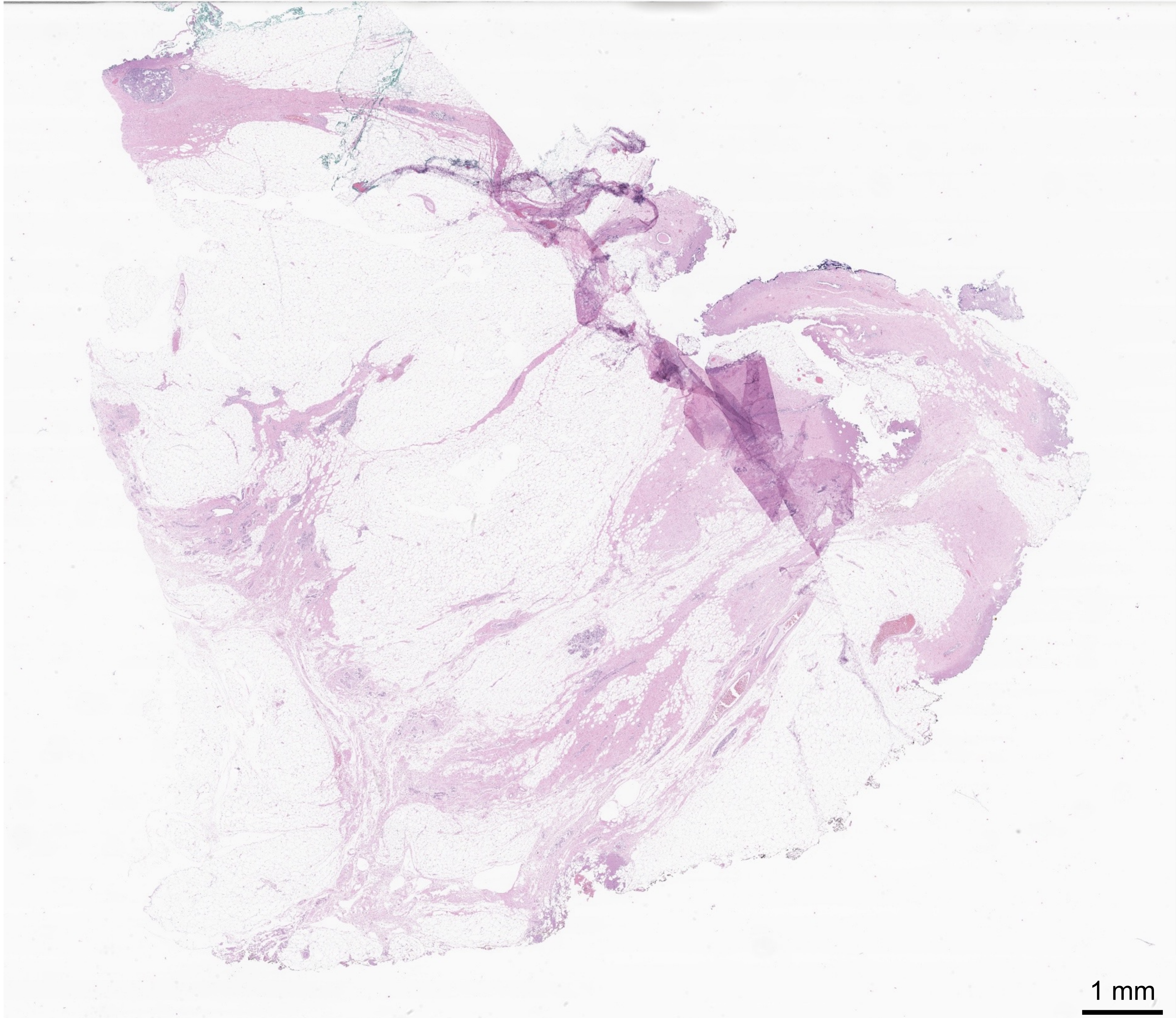

Supplementary Fig. 17 Higher resolution original images of the slides used for comparison of GrandQC with three other open-source tools for quality control (Supplementary Fig. 11-15).

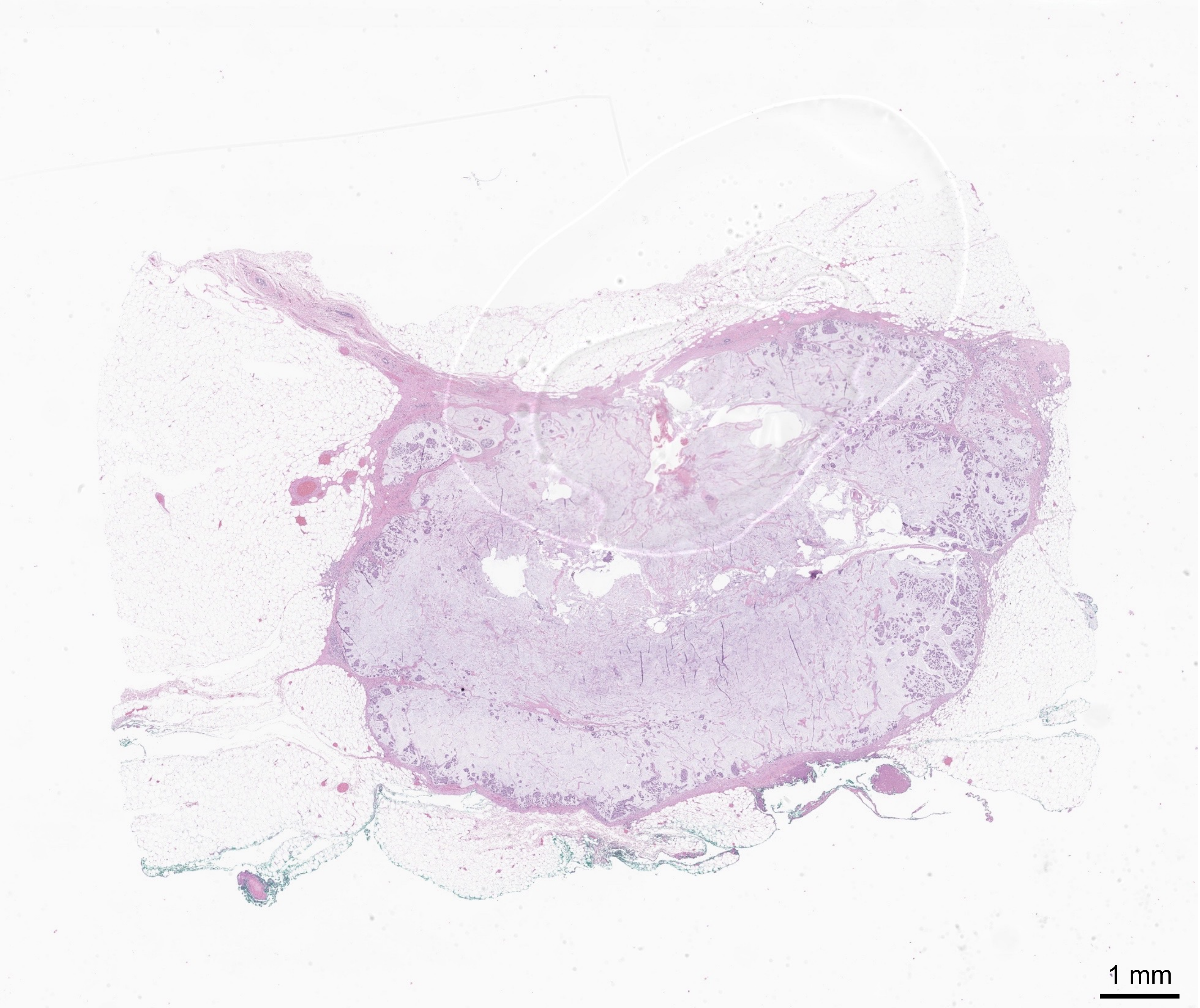

Supplementary Fig. 18 Higher resolution original images of the slides used for comparison of GrandQC with three other open-source tools for quality control (Supplementary Fig. 11-15).

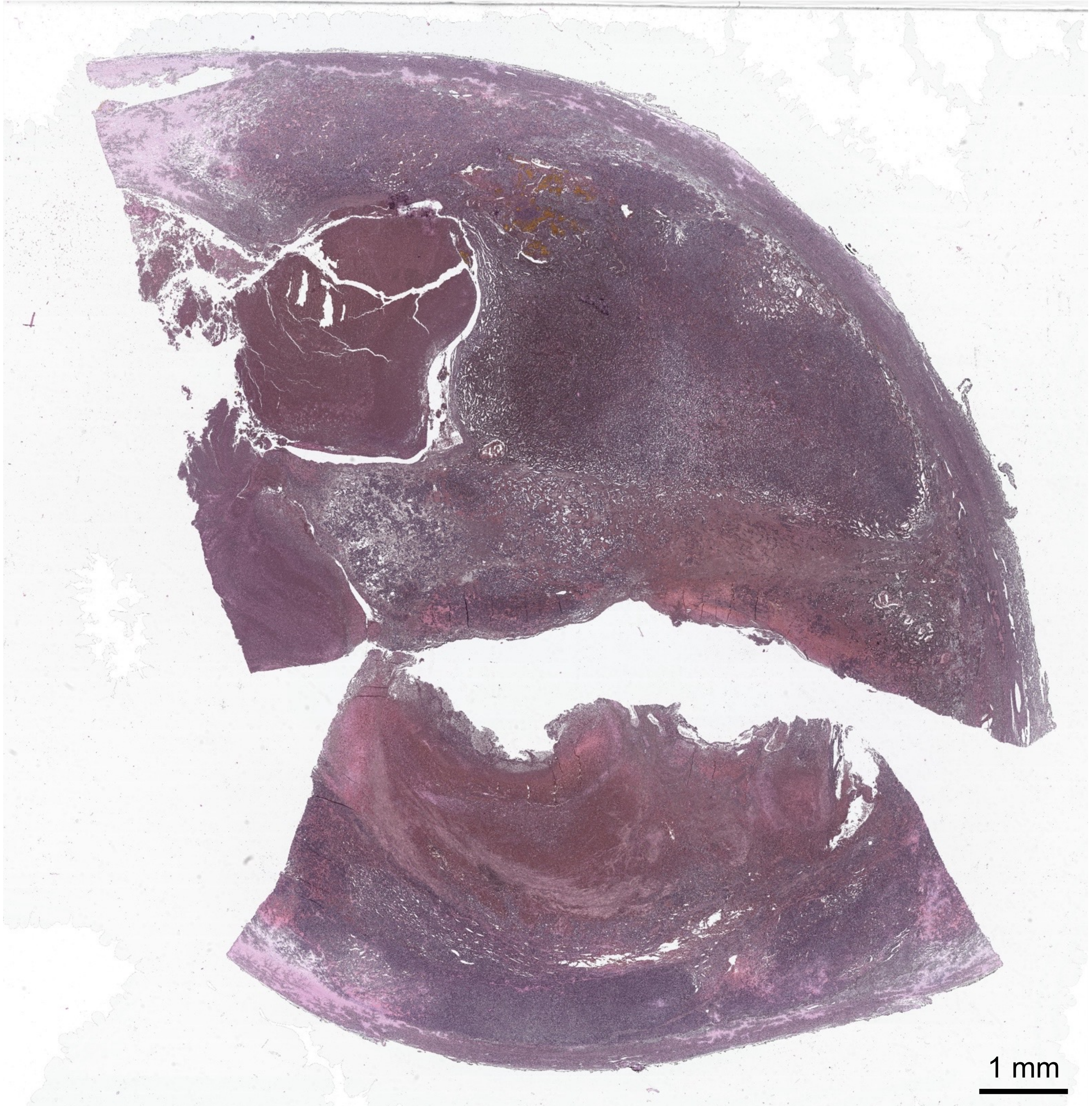

Supplementary Fig. 19 Higher resolution original images of the slides used for comparison of GrandQC with three other open-source tools for quality control (Supplementary Fig. 11-15).

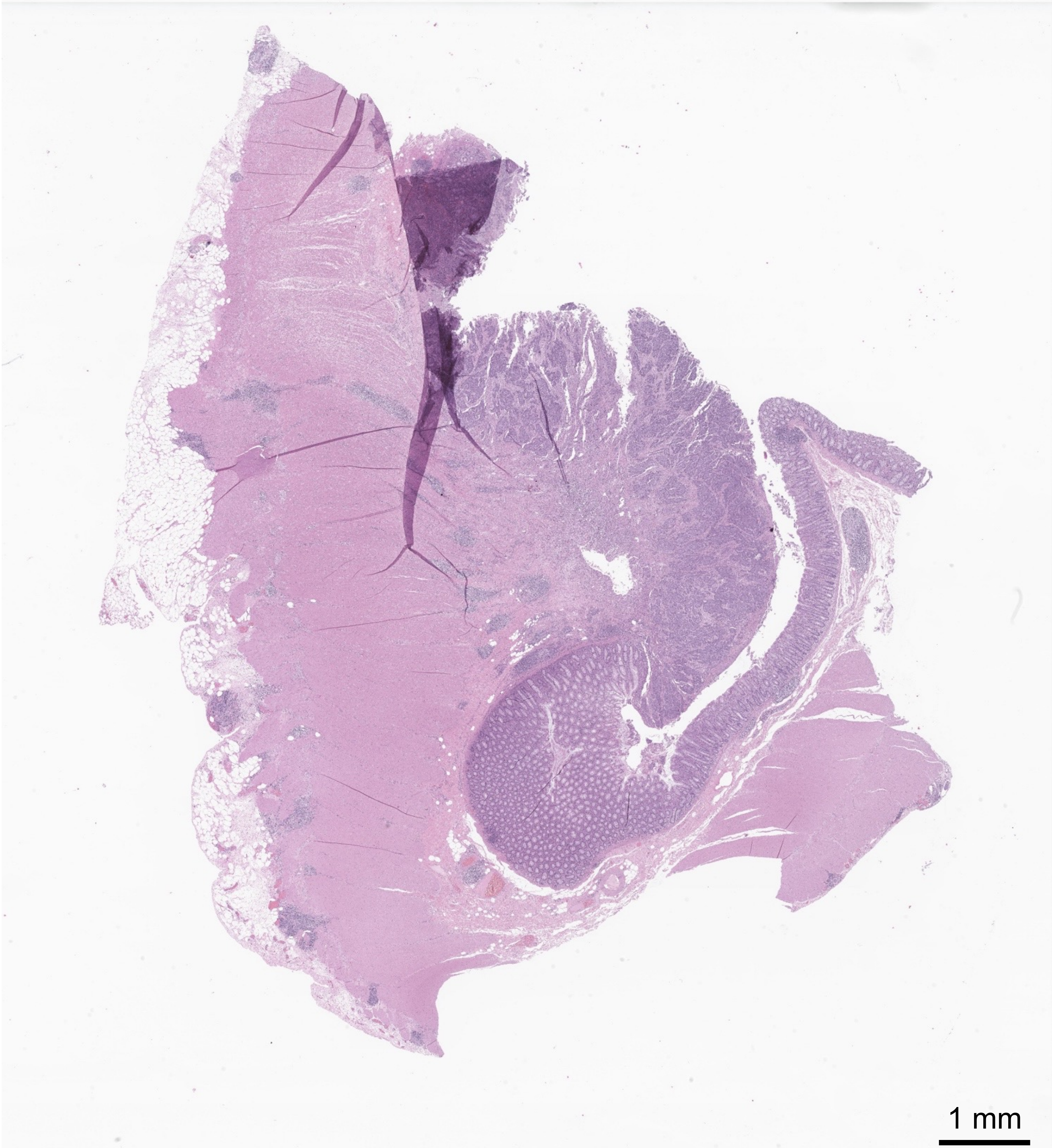

Supplementary Fig. 20 Higher resolution original images of the slides used for comparison of GrandQC with three other open-source tools for quality control (Supplementary Fig. 11-15).

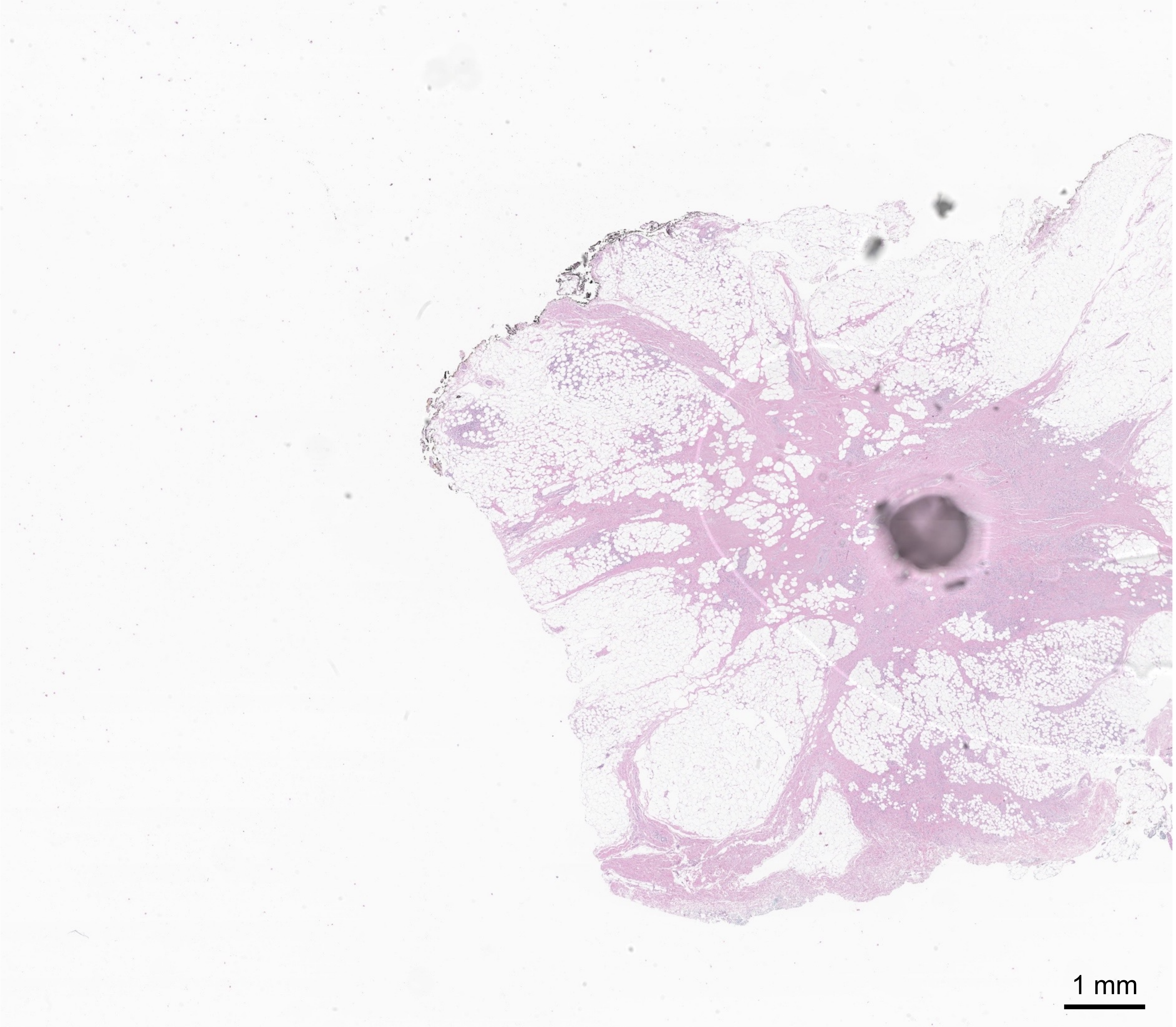

Supplementary Fig. 21 Higher resolution original images of the slides used for comparison of GrandQC with three other open-source tools for quality control (Supplementary Fig. 11-15).

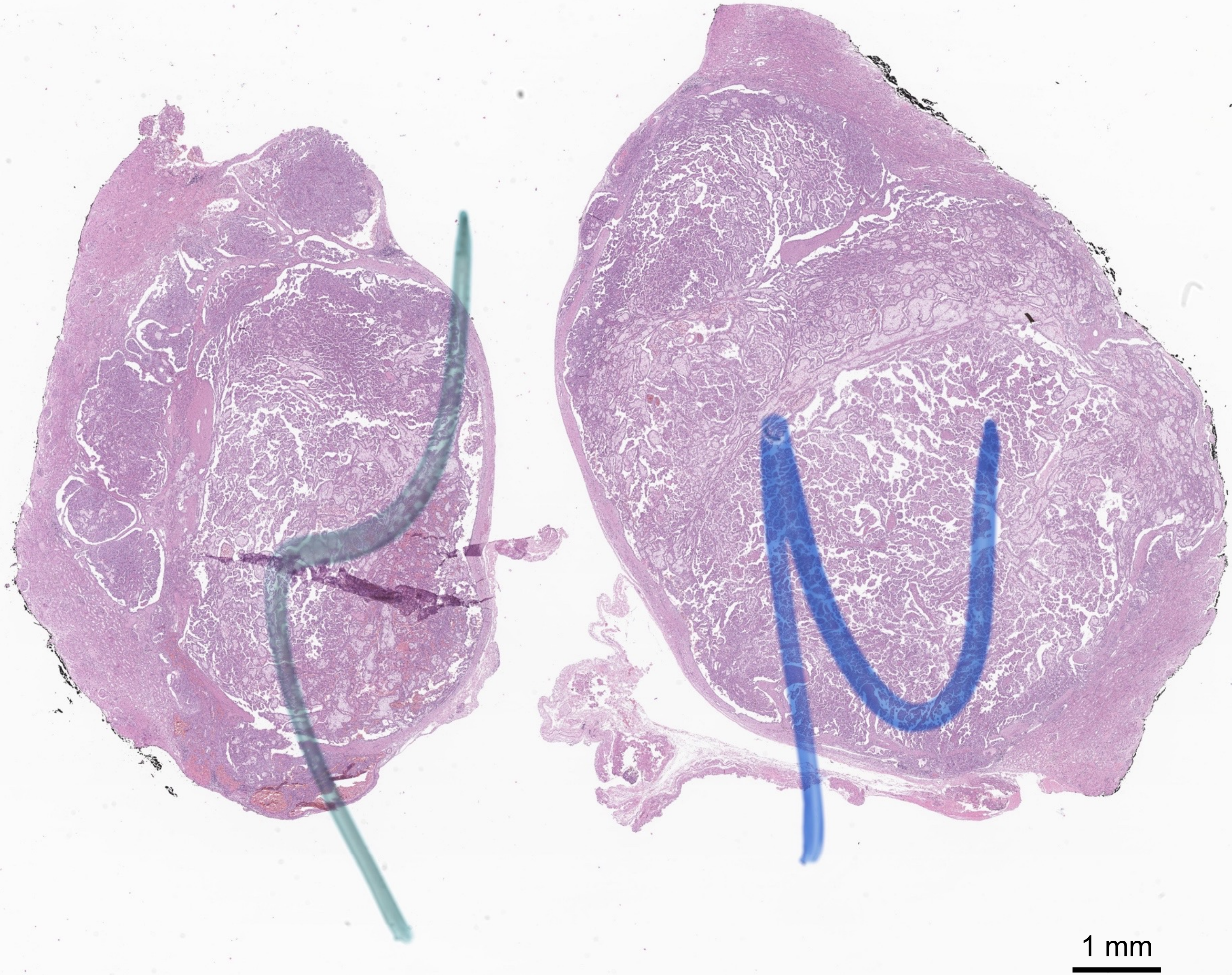

Supplementary Fig. 22 Higher resolution original images of the slides used for comparison of GrandQC with three other open-source tools for quality control (Supplementary Fig. 11-15).

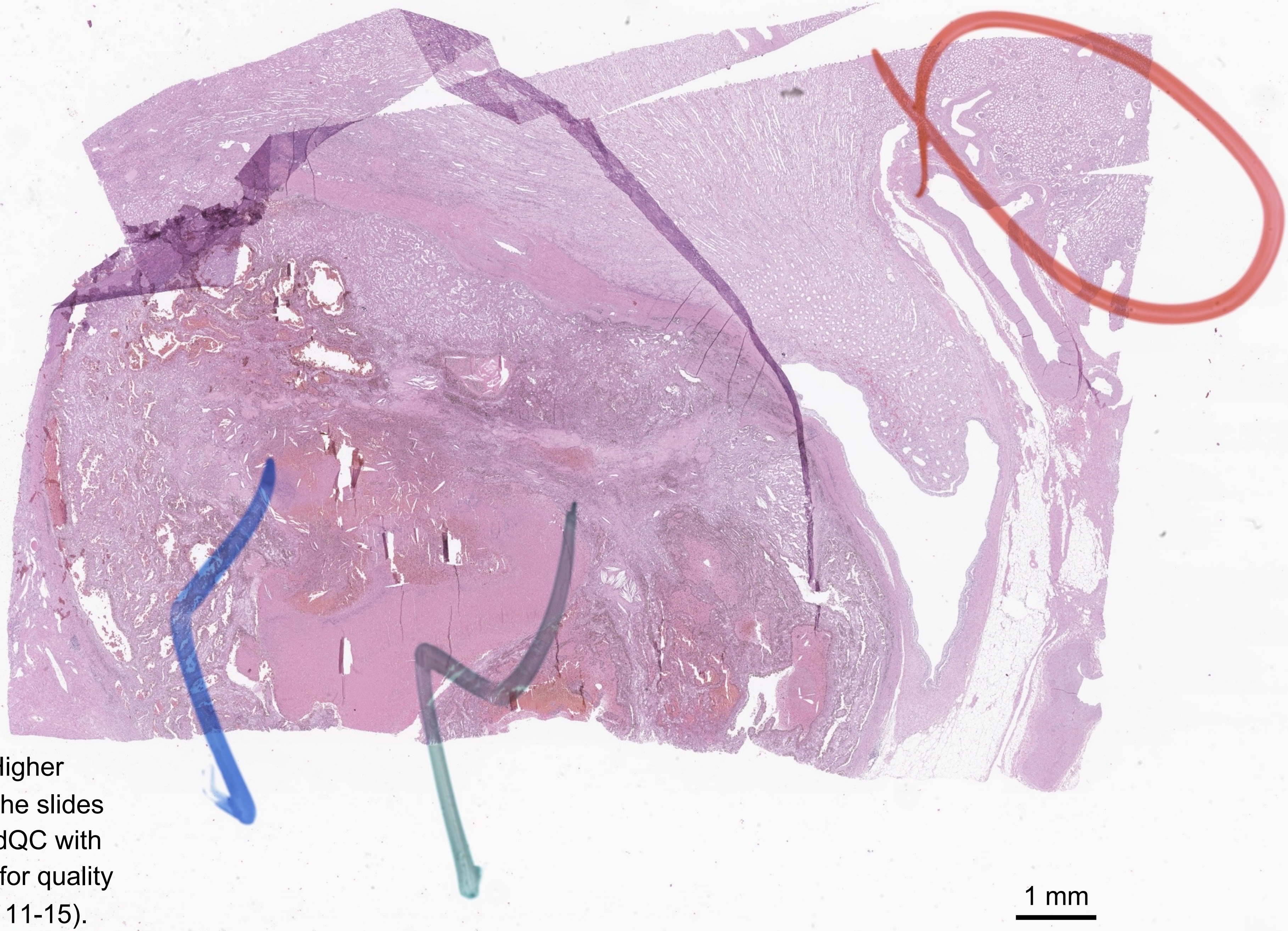

Supplementary Fig. 23 Higher resolution original images of the slides used for comparison of GrandQC with three other open-source tools for quality control (Supplementary Fig. 11-15).

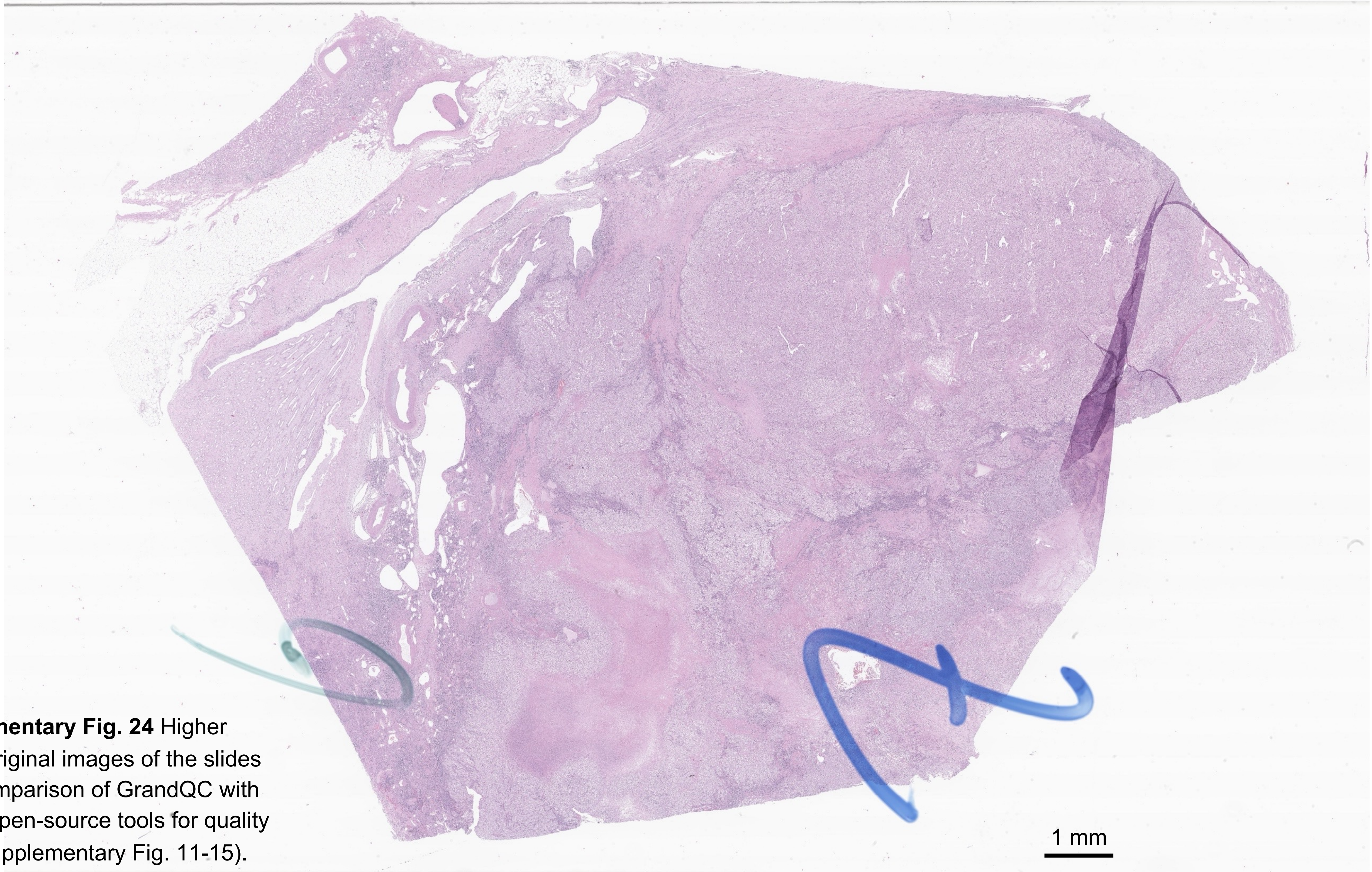

Supplementary Fig. 24 Higher resolution original images of the slides used for comparison of GrandQC with three other open-source tools for quality control (Supplementary Fig. 11-15).

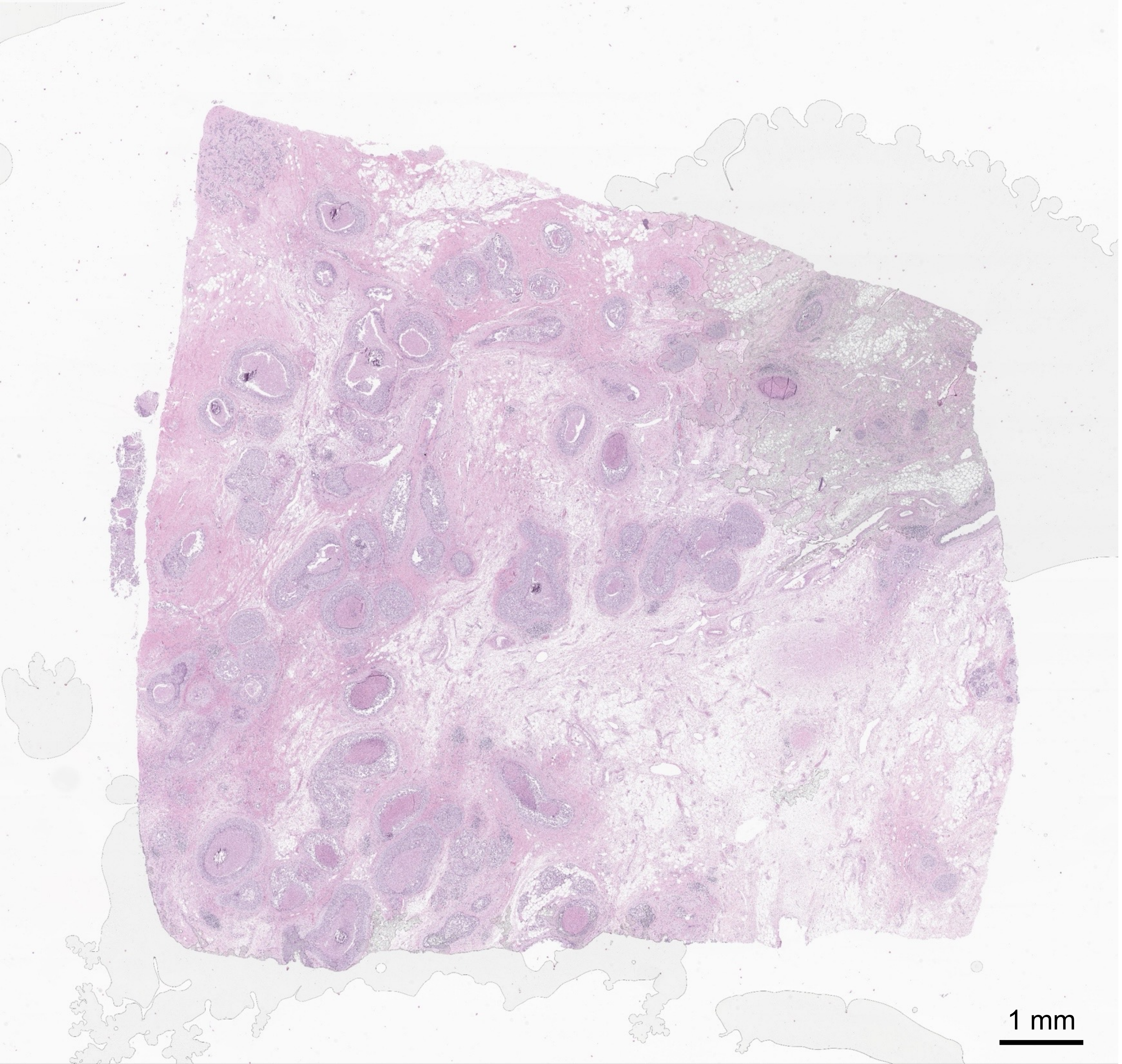

Supplementary Fig. 25 Higher resolution original images of the slides used for comparison of GrandQC with three other open-source tools for quality control (Supplementary Fig. 11-15).

Suppl. Table 1. Algorithm development steps (finding optimal architecture and hyperparameters)

Default Parameters

Epoches	Patch Size	Optimizer	Initial Learning Rate
64	512	Adam	0.0005

Comparison: Different ways to generate Out of Focus

Encoder	Decoder	MPP	Batch Size	Loss	Out of Focus Generation	Dice score of OOF	Overall Dice Score
EfficientNet-B0	UNet++	1.5	21	CrossEntropy with classes weight	Gaussian Blur with equal percentage for each kernel siz	0.849	0.754
EfficientNet-B0	UNet++	1.5	21	CrossEntropy with classes weight	80% Gaussian Blur with kernel sizes of 3 and 5	0.835	0.747
EfficientNet-B0	UNet++	1.5	21	CrossEntropy with classes weight	90% Gaussian Blur with kernel sizes of 3 and 5	**0.854**	**0.766**

Comparison: Different Encoder

Encoder	Decoder	MPP	Batch Size	Loss	Out of Focus Generation	Overall Dice Score
ResNet50	UNet	2	21	CrossEntropy with classes weight	90% Gaussian Blur with kernel sizes of 3 and 5	0.712
EfficientNet-B0	UNet	2	21	CrossEntropy with classes weight	90% Gaussian Blur with kernel sizes of 3 and 5	**0.804**
EfficientNet-B1	UNet	2	21	CrossEntropy with classes weight	90% Gaussian Blur with kernel sizes of 3 and 5	0.682
ViT	Segmenter	2	8	CrossEntropy without classes weight	90% Gaussian Blur with kernel sizes of 3 and 5	0.558

Comparison: Different Loss Function and Decoder

Encoder	Decoder	MPP	Batch Size	Loss	Out of Focus Generation	Overall Dice Score
ResNet50	UNet++	2	24	Lovasz-Softmax Loss + CrossEntropy	90% Gaussian Blur with kernel sizes of 3 and 5	0.616
ResNet50	UNet++	2	24	BoundaryLoss + CrossEntropy	90% Gaussian Blur with kernel sizes of 3 and 5	0.584
ResNet50	UNet++	2	24	0.6Focal + 0.4Dice	90% Gaussian Blur with kernel sizes of 3 and 5	0.689
ResNet50	UNet++	2	24	CrossEntropy with classes weight	90% Gaussian Blur with kernel sizes of 3 and 5	**0.728**
ResNet50	UNet++	2	24	Lovasz-Softmax Loss	90% Gaussian Blur with kernel sizes of 3 and 5	0.627
ResNet50	UNet	2	24	Focal	90% Gaussian Blur with kernel sizes of 3 and 5	0.704
ResNet50	UNet	2	24	Dice Loss	90% Gaussian Blur with kernel sizes of 3 and 5	**0.710**
ResNet50	UNet	2	24	Lovasz Loss	90% Gaussian Blur with kernel sizes of 3 and 5	0.681

Comparison: Different Batch Size and Decoder

Encoder	Decoder	MPP	Batch Size	Loss	Out of Focus Generation	Oversampling	Overall Dice Score
EfficientNet-B0	UNet++	1.5	14	CrossEntropy with classes weight	90% Gaussian Blur with kernel sizes of 3 and 5	15k	0.778
EfficientNet-B0	UNet++	1.5	21	CrossEntropy with classes weight	90% Gaussian Blur with kernel sizes of 3 and 5	15k	0.778
EfficientNet-B0	UNet++	1.5	28	CrossEntropy with classes weight	90% Gaussian Blur with kernel sizes of 3 and 5	15k	**0.788**
EfficientNet-B0	Deeplabv3+	1.5	14	CrossEntropy with classes weight	90% Gaussian Blur with kernel sizes of 3 and 5	15k	**0.810**
EfficientNet-B0	Deeplabv3+	1.5	21	CrossEntropy with classes weight	90% Gaussian Blur with kernel sizes of 3 and 5	15k	0.797
EfficientNet-B0	UNet	1.5	14	CrossEntropy with classes weight	90% Gaussian Blur with kernel sizes of 3 and 5	15k	**0.783**
EfficientNet-B0	UNet	1.5	21	CrossEntropy with classes weight	90% Gaussian Blur with kernel sizes of 3 and 5	15k	0.773

Comparison: Different Amounts of Oversampling and Decoder

Encoder	Decoder	MPP	Batch Size	Loss	Out of Focus Generation	Oversampling	Overall Dice Score
EfficientNet-B0	UNet++	1.5	14	CrossEntropy with classes weight	90% Gaussian Blur with kernel sizes of 3 and 5	10k	**0.811**
EfficientNet-B0	UNet++	1.5	14	CrossEntropy with classes weight	90% Gaussian Blur with kernel sizes of 3 and 5	13k	0.783
EfficientNet-B0	UNet++	1.5	14	CrossEntropy with classes weight	90% Gaussian Blur with kernel sizes of 3 and 5	15k	0.778
EfficientNet-B0	UNet++	1.5	21	CrossEntropy with classes weight	90% Gaussian Blur with kernel sizes of 3 and 5	10k	**0.807**
EfficientNet-B0	UNet++	1.5	21	CrossEntropy with classes weight	90% Gaussian Blur with kernel sizes of 3 and 5	15k	0.778
EfficientNet-B0	UNet++	1.5	21	CrossEntropy with classes weight	90% Gaussian Blur with kernel sizes of 3 and 5	22k	0.780
EfficientNet-B0	UNet	1.5	21	CrossEntropy with classes weight	90% Gaussian Blur with kernel sizes of 3 and 5	15k	0.794
EfficientNet-B0	UNet	1.5	21	CrossEntropy with classes weight	90% Gaussian Blur with kernel sizes of 3 and 5	22k	**0.794**

Comparison: Different Weight of Out of Focus

Encoder	Decoder	MPP	Batch Size	Loss	Out of Focus Generation	Oversampling	Weights Principle	Dice score of OOF	Overall Dice Score
EfficientNet-B0	UNet	1.5	21	CrossEntropy with classes weight	90% Gaussian Blur with kernel sizes of 3 and 5	15k	OOF * 1.1	0.856	**0.823**
EfficientNet-B0	UNet	1.5	21	CrossEntropy with classes weight	90% Gaussian Blur with kernel sizes of 3 and 5	15k	OOF * 1.2	**0.858**	0.801
EfficientNet-B0	UNet	1.5	21	CrossEntropy with classes weight	90% Gaussian Blur with kernel sizes of 3 and 5	15k	OOF * 1.3	0.857	0.765
EfficientNet-B0	UNet	1.5	21	CrossEntropy with classes weight	90% Gaussian Blur with kernel sizes of 3 and 5	15k	OOF * 1.5	0.834	0.793

Comparison: Different methods for dealing with class imbalance

Encoder	Decoder	MPP	Batch Size	Loss	Out of Focus Generation	Oversampling	Weights Principle	Overall Dice Score
EfficientNet-B0	UNet	1.5	21	CrossEntropy with classes weight	90% Gaussian Blur with kernel sizes of 3 and 5	-	-	0.766
EfficientNet-B0	UNet	1.5	21	CrossEntropy with classes weight	90% Gaussian Blur with kernel sizes of 3 and 5	15k	-	**0.794**
EfficientNet-B0	UNet	1.5	21	CrossEntropy with classes weight	90% Gaussian Blur with kernel sizes of 3 and 5	15k	Global + Oversampling	0.760
EfficientNet-B0	UNet	1.5	21	CrossEntropy with classes weight	90% Gaussian Blur with kernel sizes of 3 and 5	27k	Global + Oversampling	0.786

Final Models with different magnifications

Encoder	Decoder	MPP	Batch Size	Loss	Out of Focus Generation	Oversampling	Weights Principle	Overall Dice Score
EfficientNet-B0	UNet	2	21	CrossEntropy with classes weight	90% Gaussian Blur with kernel sizes of 3 and 5	10k	-	0.785
EfficientNet-B0	UNet	1.5	21	CrossEntropy with classes weight	90% Gaussian Blur with kernel sizes of 3 and 5	27k	Global + Oversampling	0.808
EfficientNet-B0	UNet	1	21	CrossEntropy with classes weight	90% Gaussian Blur with kernel sizes of 3 and 5	15k	-	**0.824**

Suppl. Table 2. Analysis of slides from multiple pathology departments with GrandQC

ID	Institutions	ORGAN	SCANNER	AMOUNT
P1	Large non-university pathology institute, China*	Prostate	Hamamatsu S360	600
P2	Middle-size non-university pathology institute, Austra	Multi-organ	Leica GT450	349
			Hamamatsu S360	251
P3	Large university pathology institute, USA**	Prostate	Zeiss	488
P4	Middle-size non-university pathology institute, Germany	Prostate	Leica GT450	192
P5	Large university pathology institute, Spain	Head&Neck cancer	Leica GT450	600
P6	Middle-size university pathology institute, Germany	Melanoma	Leica GT450	207
P7	Large university pathology institute, USA****	Prostate	Leica AT2	287
P8	Small non-university pathology institute, Germany	Breast	Leica GT450	99
P9	Large university pathology institute, Germany	Colon/Rectum	Leica GT450	132
P10	Middle-size non-university pathology institute, Japan	Lung	Philips	180
		Colon/Rectum		174
P11	Large university pathology institute, Netherlands***	Prostate	3DHISTECH	62
P12	Large university pathology insitute, USA	Kidney	Hamamatsu S360	274
P13	Middle-size non-university pathology institute, Germany	Prostate	Hamamatsu S360	142
P14	Large university pathology institute, Germany	Lung	3DHISTECH	351
P15	Large university pathology institute, Germany	Colon/Rectum	3DHISTECH	207
P16	Large university pathology institute, Germany	Lung	Hamamatsu S360	600
P17	Large university pathology institute, Germany	Prostate	Leica S360	558
		Colon/Rectum		42
P18	Large university pathology institute, Germany	Multi-organ	Leica AT2, CS2	600
P19	Large university pathology institute, Germany	Multi-organ	Hamamatsu S360	359
			Leica GT450	241

SCANNER		
	Hamamatsu	2226
	Leica	2819
	Philips	842
	3DHISTECH	620
	Zeiss	488

ORGAN		
	Lung	1731
	Prostate	1729
	Head&Neck cancer	600
	Melanoma	207
	Breast	99
	Colon/Rectum	555
	Kidney	274
	Mutil-organ	1800

*PAIWSIT dataset. Jin et al. PAI-WSIT: An AI Service Platform With Support for Storing and Sharing Whole-Slide Images With Metadata and Annotations. IEEE Explore 2019
**Wilkinson S et al. Nascent prostate cancer heterogeneity drives evolution and resistance to intense hormonal therapy [Data set]. The Cancer Imaging Archive.
*** PESO Dataset. Bulten et al. Epithelium segmentation using deep learning in H&E-stained prostate specimens with immunohistochemistry as reference standard. Scientific reports. 2019, 9: 864
****Madabhushi, A., & Feldman, M. (2016). Fused Radiology-Pathology Prostate Dataset (Prostate Fused-MRI-Pathology) . The Cancer Imaging Archive. doi; 10.7937/k9/TCIA.2016.tlpmr1am

Suppl. Table 3. Evaluation of segmentation accuracy for tissue detection algorithm

Number of slides from different organs with different artifacts

Validation Dataset	Colon	Breast	Prostate	Kidney	Total
**OOF / glue drops**	5	5	5	5	20
**Air bubbles**	2	2	3	3	10
**Pen markings**	2	2	3	3	10
**Folds**	15	15	15	15	60
**Total**	24	24	26	26	100

Dice Score	Colon	Breast	Prostate	Kidney
**Tissue**	0.938	0.902	0.968	0.922
**Background**	0.987	0.969	0.979	0.988

Suppl. Table 4. Comparison of HistoROI and GrandQC tools

**Dice Score**		**HistoROI**	**GrandQC**
**Colon**	Tissue w/o artifacts	0.757	0.874
	Artifacts	0.459	0.682
**Breast**	Tissue w/o artifacts	0.661	0.840
	Artifacts	0.584	0.747
**Kidney**	Tissue w/o artifacts	0.499	0.932
	Artifacts	0.215	0.546
**Prostate**	Tissue w/o artifacts	0.697	0.917
	Artifacts	0.390	0.584
**Pen markings**	Pen markings	0.818	0.984

Suppl. Table 5. Comparison of PathProfiler and GrandQC tools

Dice Score		PathProfilter	GrandQC
**Colon**	Tissue w/o artifacts	0.741	0.874
	FOLD	0.057	0.801
	OOF	0.036	0.903
	Other Artifacts	0.364	0.515
**Breast**	Tissue w/o artifacts	0.656	0.840
	FOLD	0.132	0.776
	OOF	0.043	0.908
	Other Artifacts	0.176	0.533
**Kidney**	Tissue w/o artifacts	0.644	0.932
	FOLD	0.113	0.687
	OOF	0.000	0.807
	Other Artifacts	0.091	0.482
**Prostate**	Tissue w/o artifacts	0.758	0.917
	FOLD	0.111	0.727
	OOF	0.017	0.671
	Other Artifacts	0.190	0.599
**Pen markings**	Pen markings	0.665	0.984

Suppl. Table 6. Comparison of HistoQC and GrandQC tools

	Dice Score	HistoQC	GrandQC
**Colon**	Tissue w/o artifacts	0.448	0.871
	Artifacts + background	0.122	0.716
**Breast**	Tissue w/o artifacts	0.491	0.829
	Artifacts + background	0.050	0.808
**Kidney**	Tissue w/o artifacts	0.431	0.929
	Artifacts + background	0.258	0.691
**Prostate**	Tissue w/o artifacts	0.439	0.913
	Artifacts + background	0.052	0.817
**Pen markings**	Pen markings	0.893	0.984

Supplementary methods

Annotations

Firstly, in QuPath v0.4.3 software[1], the Thresholder instrument was used to generate the annotations based on the averaged pixel values over RGB channels. Later, all annotations were checked by human experts and corrected if necessary. Most corrections involved excluding non-tissue objects (foreign objects, dark spots, glass edges) and including non-recognized tissues (mostly fatty tissue but also other tissue types in weakly stained slides). Importantly, pen markings were considered as "tissue" due to two considerations: 1) pen markings are of different transparency, with some of them non-transparent but still overlying tissue, and 2) pen markings can be easily identified and masked in the second step (artifact detection).

Background (as a separate class for artifact detection algorithm) was generated using QuPath Thresholder like the tissue detection dataset, but only in the immediate proximity of the annotated artifacts or tissue without artifacts through the inflation of their borders by approximately 200 µm. Dense annotations were created where possible.

Generation of synthetic OOF

Empirically, Gaussian blur produced better results in initial tests compared to other methods (e.g., Bokeh) and was selected as the main strategy. Generation of OOF was performed on patches extracted from annotated regions, only in areas annotated as tissue without artifacts. Using approximately 30,000 unique patch-level binary masks (reusing masks for one of the classes, benign prostate tissue, from another project[2]), representative of different forms and sizes of objects and spatial locations in the patch, the OOF was generated in part of each patch according to the binary map so that every such image patch has transition regions between OOF and non-artificially changed tissue. The training patches were shuffled before OOF application, and OOF was generated until the OOF area reached 30% of the non-artificially changed tissue in the whole training dataset. For OOF generation with Gaussian blur, the probability distribution for kernel sizes was set to be 90% for kernels 3 and 5, corresponding to the slight focus deterioration more common in WSIs.

Algorithm development

Magnification, tile size, learning rate, batch size, data augmentation, and class weighting were all considered as hyperparameters and tested in a broad range using a fine-tuning/validation subset (approximately 10% of the training dataset for both tasks). During the training phase, addressing the class imbalance issue arising from varying frequencies and sizes of annotated areas of different artifacts was pivotal. We prioritized two primary solutions: class weight during loss function quantification, oversampling of underrepresented classes, and batch engineering. For tissue detection, global class weights proved to be a reliable solution, while for artifact detection, the best results in the validation dataset were achieved with a setup combining aggressive oversampling, global weights, and batch engineering using an equal number of patches for different classes within single batches. For artifact detection tasks, the algorithms were trained using three different magnification levels (roughly 5x, 7x, and 10x) to assess in detail the trade-offs between precision and analysis speed. All algorithms were developed in Python 3.9 using the Pytorch framework (v.1.10), the segmentation-models-pytorch package for algorithm construction, custom training scripts, and the albumentations package for data augmentation. Brightness, contrast, hue, saturation, flips/rotations, JPEG compression, and Gaussian noise were used for data augmentation.

Hardware

Algorithm training and validation was performed using A100 80 Gb and H100 32Gb NVIDIA GPU cards. The performance analysis was performed on a high-end PC station with NVIDIA RTX 3090 24 Gb graphic card. In the departments of FED-PATH consortium (University Hospitals Essen, Leipzig, Halle), the test experiments were performed in a decentralized mode using PC stations equipped with consumer-grade GPU cards.

References

1. Bankhead, P., Loughrey, M. B., Fernández, J. A., Dombrowski, Y., McArt, D. G., Dunne, P. D. et al. QuPath: Open source software for digital pathology image analysis. *Scientific Reports 2017 7:1* **7**, 1–7 (2017).
2. Tolkach, Y., Dohmgörgen, T., Toma, M. & Kristiansen, G. High-accuracy prostate cancer pathology using deep learning. *Nat Mach Intell* **2**, 411–418 (2020).
