# Supplementary material for: GrandQC: A comprehensive solution to quality control problem in digital pathology
Source: Nat Commun. 2024 Dec 16;15:10685. doi: 10.1038/s41467-024-54769-y (PMC11649692; doi:10.1038/s41467-024-54769-y)
Supplement: Supplementary file 2 — Reporting Summary [file 41467_2024_54769_MOESM2_ESM.pdf]

nature portfolio

Corresponding author(s): Yuri Tolkach

Last updated by author(s): Nov 9, 2024

Reporting Summary

Nature Portfolio wishes to improve the reproducibility of the work that we publish. This form provides structure for consistency and transparency in reporting. For further information on Nature Portfolio policies, see our Editorial Policies and the Editorial Policy Checklist.

Statistics

For all statistical analyses, confirm that the following items are present in the figure legend, table legend, main text, or Methods section.

n/a	Confirmed	
☐	☒	The exact sample size (n) for each experimental group/condition, given as a discrete number and unit of measurement
☒	☐	A statement on whether measurements were taken from distinct samples or whether the same sample was measured repeatedly
☒	☐	The statistical test(s) used AND whether they are one- or two-sided<br>*Only common tests should be described solely by name; describe more complex techniques in the Methods section.*
☒	☐	A description of all covariates tested
☒	☐	A description of any assumptions or corrections, such as tests of normality and adjustment for multiple comparisons
☐	☒	A full description of the statistical parameters including central tendency (e.g. means) or other basic estimates (e.g. regression coefficient) AND variation (e.g. standard deviation) or associated estimates of uncertainty (e.g. confidence intervals)
☒	☐	For null hypothesis testing, the test statistic (e.g. F, t, r) with confidence intervals, effect sizes, degrees of freedom and P value noted<br>*Give P values as exact values whenever suitable.*
☒	☐	For Bayesian analysis, information on the choice of priors and Markov chain Monte Carlo settings
☒	☐	For hierarchical and complex designs, identification of the appropriate level for tests and full reporting of outcomes
☒	☐	Estimates of effect sizes (e.g. Cohen's d, Pearson's r), indicating how they were calculated

Our web collection on statistics for biologists contains articles on many of the points above.

Software and code

Policy information about availability of computer code

Data collection	The data management and processing was performed using QuPath (qupath.github.io) software.
Data analysis	Training of algorithms was performed using Python 3.9. and pytorch (v.2.0.1) framework. Segmentation models pytorch (0.3.1) library was used to create the algorithms. Data analysis was performed using Python 3.9 and R v. 3.4. For whole-slide image processing openslide library (v 3.4.1) was used.

For manuscripts utilizing custom algorithms or software that are central to the research but not yet described in published literature, software must be made available to editors and reviewers. We strongly encourage code deposition in a community repository (e.g. GitHub). See the Nature Portfolio guidelines for submitting code & software for further information.

Data

Policy information about availability of data

All manuscripts must include a data availability statement. This statement should provide the following information, where applicable:

- Accession codes, unique identifiers, or web links for publicly available datasets
- A description of any restrictions on data availability
- For clinical datasets or third party data, please ensure that the statement adheres to our policy

The datasets and models generated or used in this study have been made available with following ways: QC masks generated from the TCGA public dataset are deposited at Zenodo under accession code (https://zenodo.org/records/14041578). The test datasets (image patches with corresponding ground truth maps of manual annotations), stratified by organ are deposited at Zenodo under accession code (https://zenodo.org/records/14039591). The whole-slide images from TCGA

cohort are available from https://portal.gdc.cancer.gov/. PESO dataset is available from https://zenodo.org/records/1485967. Wilkinson et al. dataset and Madabhushi et al. datasets are available from The Cancer imaging archive (https://www.cancerimagingarchive.net/). Proprietary data is not being released due to authors institutions' data privacy regulation. GrandQC is being open-sourced (https://github.com/cpath-ukk/grandqc) for academic research and non-commercial use by pathology departments only. The code is also deposited at Zenodo under accession code (https://zenodo.org/records/14062356). The use for commercial purposes is not allowed. The trained model checkpoints for three different magnifications are deposited at Zenodo under accession code (https://zenodo.org/records/14041538).

Research involving human participants, their data, or biological material

Policy information about studies with human participants or human data. See also policy information about sex, gender (identity/presentation), and sexual orientation and race, ethnicity and racism.

Reporting on sex and gender	n/a
Reporting on race, ethnicity, or other socially relevant groupings	n/a
Population characteristics	n/a
Recruitment	n/a
Ethics oversight	All study steps were performed in accordance with the Declaration of Helsinki. This study was approved by the Ethical committee of the University of Cologne, University of Essen, Medical Faculty of Martin Luther University Halle-Wittenberg, University Hospital Leipzig, Charité (joint 22-1233, Project FED-PATH; joint Cologne/Charité 20-1583), the Ethical committee of Lower Austria (GS1-EK-4/694-2021), Kameda Hospital (22-094), University Hospital Aachen (EK 405/2). The need for patient consent was waived as only anonymized, retrospective materials were used.

Note that full information on the approval of the study protocol must also be provided in the manuscript.

Field-specific reporting

Please select the one below that is the best fit for your research. If you are not sure, read the appropriate sections before making your selection.

[x] Life sciences [ ] Behavioural & social sciences [ ] Ecological, evolutionary & environmental sciences

For a reference copy of the document with all sections, see nature.com/documents/nr-reporting-summary-flat.pdf

Life sciences study design

All studies must disclose on these points even when the disclosure is negative.

Sample size	Given the nature of the study no statistical method was used to predetermine sample size.
Data exclusions	No data were excluded from the analyses.
Replication	Not applicable.
Randomization	The experiments were not randomized.
Blinding	The Investigators were not blinded to allocation during experiments and outcome assessment.

Reporting for specific materials, systems and methods

We require information from authors about some types of materials, experimental systems and methods used in many studies. Here, indicate whether each material, system or method listed is relevant to your study. If you are not sure if a list item applies to your research, read the appropriate section before selecting a response.

Materials & experimental systems

n/a	Involved in the study	
[x]	[ ]	Antibodies
[x]	[ ]	Eukaryotic cell lines
[x]	[ ]	Palaeontology and archaeology
[x]	[ ]	Animals and other organisms
[x]	[ ]	Clinical data
[x]	[ ]	Dual use research of concern
[x]	[ ]	Plants

Methods

n/a	Involved in the study	
[x]	[ ]	ChIP-seq
[x]	[ ]	Flow cytometry
[x]	[ ]	MRI-based neuroimaging

Plants

Seed stocks	*Report on the source of all seed stocks or other plant material used. If applicable, state the seed stock centre and catalogue number. If plant specimens were collected from the field, describe the collection location, date and sampling procedures.*
Novel plant genotypes	*Describe the methods by which all novel plant genotypes were produced. This includes those generated by transgenic approaches, gene editing, chemical/radiation-based mutagenesis and hybridization. For transgenic lines, describe the transformation method, the number of independent lines analyzed and the generation upon which experiments were performed. For gene-edited lines, describe the editor used, the endogenous sequence targeted for editing, the targeting guide RNA sequence (if applicable) and how the editor was applied.*
Authentication	*Describe any authentication procedures for each seed stock used or novel genotype generated. Describe any experiments used to assess the effect of a mutation and, where applicable, how potential secondary effects (e.g. second site T-DNA insertions, mosiacism, off-target gene editing) were examined.*
